# Comparative whole genome re-sequencing analysis in upland New Rice for Africa: insights into the breeding history and respective genome compositions

**DOI:** 10.1186/s12284-018-0224-3

**Published:** 2018-05-15

**Authors:** Naoki Yamamoto, Richard Garcia, Tomohiro Suzuki, Celymar Angela Solis, Yuichi Tada, Ramaiah Venuprasad, Ajay Kohli

**Affiliations:** 1 International Rice Research Institute, Los Baños, Laguna Philippines; 2 Utsunomiya University, 350 Mine-machi, Utsunomiya, Tochigi Japan; 3 Tokyo University of Technology, 1404-1 Katakura, Hachioji, Tokyo Japan; 4 Africa Rice Center, 01 BP 4029, Abidjan 01, Côte d’Ivoire

**Keywords:** Genome structure, *Oryza glaberrima*, Rice, Polymorphism, Upland NERICA, WAB56–104

## Abstract

**Background:**

Increasing rice demand is one of the consequences of the steadily improving socio-economic status of the African countries. New Rice for Africa (NERICA), which are interspecific hybrids between Asian and African rice varieties, are one of successful breeding products utilizing biodiversity across the two different rice crop species. Upland NERICA varieties (NU) exhibit agronomic traits of value for the harsh eco-geography, including shorter duration, higher yield and stress tolerance, compared to local African varieties. However, the molecular basis of the traits in NU varieties is largely unknown.

**Results:**

Whole genome re-sequencing was performed of four NU lines (3, 4, 5, and 7) and for the parental *Oryza sativa* WAB56–104 and *Oryza glaberrima* CG14. The *k*-mer analysis predicted large genomes for the four NU lines, most likely inherited from WAB56–104. Approximately 3.1, 0.10, and 0.40 million single nucleotide polymorphisms, multi nucleotide polymorphisms, and short insertions/deletions were mined between the parental lines, respectively. Integrated analysis with another four NU lines (1, 2, 8, and 9) showed that the ratios of the donor CG14 allelic sites in the NU lines ranged from 1.3 to 9.8%. High resolution graphical genotype indicated genome-level similarities and common genetic events during the breeding process: five *xyloglucan fucosyltransferase* from *O. glaberrima* were introgressed in common. Segregation of genic segments revealed potential causal genes for some agronomic traits including grain shattering, awnness, susceptibility to bacterial leaf bright, and salt tolerance. Analysis of unmapped sequences against the reference cultivar Nipponbare indicated existence of unique genes for pathogen and *a*biotic stress resistance in the NU varieties.

**Conclusions:**

The results provide understanding of NU genomes for rice improvement for Africa reinforcing local capacity for food security and insights into molecular events in breeding of interspecific hybrid crops.

**Electronic supplementary material:**

The online version of this article (10.1186/s12284-018-0224-3) contains supplementary material, which is available to authorized users.

## Background

Changing socio-economic conditions are driving the progressively increasing consumption of rice in the African countries. Hence, African countries have imported more than 10 million tons of rice annually in recent years (FAO 2013a). Besides, there is a sustained effort to supplement the imports with local rice cultivation in Africa. Namely, yield increase and harvested area expansion both were achieved to pull up the total rice production in Africa since 2000 (FAO 2013b). To further facilitate those, the improvement of the locally suited rice varieties is an important aspect in rice breeding.

Upland rice production is feasible with labor-saving management and can be a source of food and income in Africa. To integrate rice cultivation in poverty and hunger reduction, upland farmers should be able to grow rice. The development of the first generation upland NERICA (NU) varieties by the West Africa Rice Development Association (WARDA: at present the Africa Rice Center) in the 1990s was an important step in that direction. NU varieties are the products of surmounting above the reproductive barriers between Asian rice *Oryza sativa* and African rice *Oryza glaberrima* Steud. (Sano 1990). One of the first subsets of NU varieties (NU 1 to 11), derived from an upland tropical *japonica* cultivar WAB56–104,developed by WARDA as the recurrent parent, and the *O. glaberrima* accession CG14 as the donor parent, were released in 2000s (Sié et al. 2012). These NUs were adapted into the upland rice ecosystems in West African countries, exhibiting good yield potential (4–5 t/ha), short duration, no grain shattering and tolerance to lodging (Africa Rice Center 2008; Somado et al. 2008). Moreover, the NU varieties exhibit superior tolerance of biotic and *a*biotic stress factors such as rice yellow mottle virus (Attere and Fatokun 1983; Albar et al. 2003; Paul et al. 2003), rice blast (Somado et al. 2008), stem borders (Rodenburg et al., 2015), soil acidity (Somado et al. 2008), and soil salinity (Awala et al. 2010). Arguably, the NU rice grains exhibit high protein content (~ 10%) which makes for better nutritious value for the African consumers (Somado et al. 2008).

The NU varieties would be useful not only as maternal lines in rice breeding but also intermediary breeding materials that can transfer genes underlying useful agronomic traits from the parental lines into other varieties. The donor species *O. glaberrima* has attracted attention as a source of biotic and *a*biotic stress tolerance genes: i.e. genes for bacterial leaf blight, cyst nematode, iron toxicity, drought and weed competitiveness (Sano et al. 1984; Jones et al. 1997; Lorieux et al. 2003; Haefele et al. 2004; Singh et al. 2004; Sarla and Swamy 2005; Majerus et al. 2007; Vikal et al. 2007). In addition, the recurrent *O. sativa* parents seem to have useful genes for African rice breeding because those were selected from hundred lines under the local climate (Jones et al. 1997). Traore et al. (2011) reported that WAB56–104 showed long grain phenotype (~ 7.7 mm), low amylase content, and relatively short duration. Recently, a draft genome sequence of the donor parent of *O. glaberrima* accession CG14 was reported (Wang et al. 2014), however, there is a lack of genetic information on NU and the recurrent parental varieties.

Understanding on genome architecture of the NU varieties would promote their appropriate and informed utilization in rice breeding for African countries. However, it is rather vague if deciphered from the existing genomic profiles. Genotyping by the limited number of DNA markers provided evidence for macro-level similarity among NU genomes and also some non-parental alleles (Semagn et al. 2007; Fukuta et al. 2012; Pariasca-Tanaka et al. 2015). Recently, four NU varieties (1, 2, 8 and 9) were sequenced at 10 to 15X depth as a part of the rice 3000 genome project (Li et al. 2014; Alexandrov et al. 2015). An in-depth analysis of their genome sequences remains to be achieved.

In the present study, we conducted comparative re-sequencing analysis of the NU genomes using the Illumina high-throughput sequencing technique. We re-sequenced the genomes of four additional NU varieties (3, 4, 5, and 7) as well as of the two parental lines. Genome size and homozygosity were examined by *k*-mer analysis of the sequencing data. We then performed an integrated sequence analysis of all eight NU varieties for single nucleotide polymorphisms (SNPs), multi nucleotide polymorphisms (MNPs) and short insertion and deletions (InDels). Application of the whole genome polymorphisms allowed prediction and characterization of the NU genome structures at high resolution. Enrichment of Gene Ontology on common and variety/lineage-specific introgression segments from CG14 was tested for biased gene distributions. Analysis of chromosomal segment patterns identified gene candidates that are associated with phenotype. Gene information from WAB56–104 unmapped sequences, which were not mapped on the Nipponbare genome sequence properly, were analyzed and integrated with the four NU varieties sequenced in this study.

## Methods

### Whole genome sequencing

Rice seeds were distributed from Africa Rice Center (Côte d’Ivoire). Genomic DNA was prepared from etiolated seedlings cultured in the half strength of Murashige & Skoog liquid medium (Murashige and Skoog 1962). DNA extraction was performed using the method described by Doyle (1987) with a few modifications. Integrity of DNA was analyzed by electrophoresis in 2% agarose, and DNA samples were subjected to high-throughput sequencing by Illumina HiSeq 4000 in Macrogen Inc. (Seoul, Korea). In the sequencing analysis, DNA library was constructed from fragmented genomic DNA (ranging from approx. 0.2 to 10 kb) using TruSeq DNA PCR-FREE (350 bp) (Illumina), and 101 bp paired-end short reads were generated in fastq format. The obtained raw data were processed by the Trimmomatic software version 0.33 (Bolger et al. 2014) with the options of ‘LEADING:15, TRAINLING:15, SLIDINGWINDOW:4:15, MINLEN:20’ to trim adapter and low-quality sequences. The raw sequencing data were deposited to DDBJ Sequencing Read Archive (accession number: DRA006795).

### *k*-mer analysis

The pre-processed high quality reads were applied to count *k*-mers by the software KMC 2 version 2.3.0 (Deorowicz et al. 2015). Genome size prediction was carried out using the software GCE version 1.0.0, which utilizes a Bayes model based method (Liu et al. 2013). Reference data were downloaded from NCBI Sequencing Read Archive for cv. Nipponbare (SRR545231, SRX179262, DRR028131, DRR028132, DRR083658, and SRR1043564), IR64 (SRR3098100), and Moroberekan (DRR003661).

### Flow cytometry analysis

Genome DNA contents (pg/2C) were determined by a flow cytometry analyzer EC800 (Sony Biotechnology Inc., CA, USA). Nuclei of samples were prepared as follows: 1) fresh leaves were chopped into finely minced tissues using a razor blade and mixed with 600 μl of extraction buffer [50 mM Tris-HCl (pH 7.5), 0.5% polyvinypyrrolidone-K90, 0.01% Triton X-100, and 0.63% Na_2_SO_4_] at room temperature, 2) the supernatant was filtered via a single layer of nylon mesh with 25 μm pore size (NYTAL P-25, SEFAR AG, Ruschlikon, Switzaland), 3) the filtrate was subjected to RNase treatment and staining by propidium iodide (PI) with 3 μl of RNase A (10 mg/ml) and 6 μl of Propidium Iodide (PI) Solution (0.5 mg/ml, Sony Biotechnology) for 30 min at room temperature under dark. Signals of PI-stained nuclei were obtained by the flow cytometer with a band pass filter which transmits 570 ~ 620 nm excitation of PI at 40 μl/min of flow rate. At least 1000 nuclei were analyzed per measurement. Peak areas of given signals were used for calculation of DNA contents. Each measurement was with technical duplicates and four biological replicates. A linear standard curve was made based on *Arabidopsis thaliana* (Col-0), *Brachypodium distachyon* (L.) Beauv., *Solanum lycopersicum* (cultivar Micro-Tom), and *Zea mays* (cultivar Peter corn). DNA contents of these samples were defined as 0.32, 0.70, 2.0, 5.3 pg/2C, respectively, based on prior knowledge (Arumuganathan and Earle 1991; Bennett et al. 2003; Bennett and Leitch 2005). Determined DNA contents were converted into haploid genome size using the conversion factor of 1 pg DNA = 978 million base pairs (Doležel et al. 2003). The rice reference cultivar Nipponbare was analyzed as a control for the African varieties.

### Bioinformatics pipeline for polymorphism mining

In order to mine polymorphism candidates, we prepared a bioinformatics pipeline. Processed sequence reads were mapped against the rice genome (16 pseudomolecules of cultivar Nipponbare, chromosome (Chr) 1 to 12, ChrUn, ChrSy, plastid, and mitochondrial genome contigs of MSU 7.0: http://rice.plantbiology.msu.edu/) by Burrows-Wheeler Aligner MEM (Li and Durbin 2009) ver. 0.7.12 with option of ‘–O 4′. This mapping condition was determined after comparisons under conditions with different gap penalty in a small batch pre-analysis. Utilization of the full set of pseudomolecules made it possible to reduce mismapping between homologous loci between pseudomolecules. Generated mapping data file (SAM file) was processed for filtering out discordant paired reads using in-house Perl scripts. Filtered SAM file was converted into a BAM file and sorted by SAMtools ver. 0.1.19 (Li et al. 2009). Sorted BAM files of 10 genotypes were co-realigned by the Genome Analysis Tool Kit ver. 3.5 (McKenna et al. 2010). Resultant mapping data were converted into a pileup format file by SAMtools with the command ‘mpileup’ with ‘–Q 0′ option. Sequence variants between the parental lines and NERICA to either parental line were called if the read depth was no less than 20 for WAB 56–104, CG 14, and NERICA 7, 25 for NERICA 5, 21 for NERICA 4, 6 for NERICA 9, 5 for NERICA 1, 18 for NERICA 3, 4 for NERICA 2 and NERICA 8 and coincidence within genotype was no less than 80% for homozygous polymorphism mining. SNPs and MNPs were called using an in-house Perl script ‘SNiPer2’. Short Indel sites were called using the software VarScan2 version 2.3.9 with the command of somatic and the options of ‘--min-base-qual 0′.

### Cleaved amplified polymorphic sequences (CAPS) assay

Genomic DNA surrounding SNP candidates were amplified by PCR using KAPA3G Plant PCR Kit (KapaBiosystems, Inc.). PCR products were digested by a restriction enzyme and electrophoresed in agarose gel with presence of FastStart SYBR Green Master (Roche Diagnostics GmbH) at 100 V for 60 min. Gels were imaged using a Molecular Imager Gel Dox XR System (Bio-Rad Laboratories, Inc.).

### Survey for structural variations

A structural variant detection tool Manta version 1.1.1 (Chen et al. 2016) was employed for calling translocation breakends, inversions, tandem repeats, long insertions and long deletions with the default condition. Raw data files (VCF format) were converted into the BEDPE format using SVtools (https://github.com/ctsa/svtools) for analysis. Potential structural variants after QC filtering were selected. Copy number variations (CNVs) were searched using CNVnator version 0.3.3 (Abyzov et al. 2011).

### Prediction of chromosomal segment type

All the SNPs, MNPs, and short InDel sites were aligned into a data matrix. Potential de novo mutation sites, which showed the same allele between the parental varieties but a different common allele in NU varieties, were excluded for reducing artifacts. Genetic imputation was carried out to infer chromosomal segments derived from WAB56–104, CG14, or non-parental varieties.

### Gene set enrichment analysis

Gene Ontology enrichment was examined by the agriGO2 web tool (Tian et al. 2017). ‘Singular Enrichment Analysis’ was applied with the default parameters. Statistical analysis options of hypergeometric test with multi test adjustment method of Holm were selected.

### De novo assembly

Full-length of unmapped paired end reads of WAB56–104 were collected and probable contaminant sequences were excluded based on results of BLASTN against human, *E. coli* and yeast genomic sequences (98% identity of 101 bp). Resultant paired-end reads were applied to de novo assembly using SOAPdenovo2 version 2.40 (Luo et al. 2012) with different *k*-mer from 21 to 81. The assembly result with the longest N50 length was obtained with *k*-mer of 67. Then, series of ambiguous sequence ‘N’ of the genomic scaffolds were filled using the software the GapCloser module version 1.12 (Luo et al. 2012).

### Gene prediction and annotation

Repeat sequences on genome scaffolds were masked using RepeatMasker version open-4.0.6 with rmblastn version 2.6.0, RepBase Update 20,160,829 and RM database version 20,160,829. Then, structural gene annotations were given using Augustus web interface (http://bioinf.uni-greifswald.de/augustus/submission.php; Keller et al. 2011). Predicted protein sequences were annotated based on BLAST searches (Blast+ version 2.4.0) against the manually annotated protein database Swiss-Prot (Boeckmann et al. 2005), all proteins in rice (MSU7), in Arabidopsis (TAIR10, Lamesch et al., 2012), in maize (maizeGDB AGPv4, Andorf et al. 2015), in *Sorghum bicolor* (PlantGDB the number 79, Duvick et al. 2008), and in Brachypodium (PlantGDB the number 192). GO terms were assigned using BLAST2GO version 4.1.9 (Conesa and Götz 2008).

### Mapping of unmapped short reads

Unmapped paired end reads were collected and mapped upon the genomic scaffolds of unmapped sequences from WAB56–104 using BWA mem with the default condition. After filtering out discordant and multi-mapped paired reads using in-house Perl scripts, averaged read depth on each gene transcribed region was calculated in each genotype.

## Results and discussion

### Whole genome re-sequencing

Re-sequencing data for NU3, 4, 5, and 7, and for WAB56–104 and CG14 were obtained at 32.5–53.8X coverage. In total 1.48 billion short reads with the length of 101 bp were generated (Additional file 1: Table S1). Analysis of the sequence reads revealed *k*-mer distributions suggesting homozygosity of the sequenced genomes (Additional file 2: Figure S1); a small peak (*k*-mer depth = 16) was observed in NERICA 5, and it could be contaminants in the sequencing steps. Genome sizes were predicted using the *k*-mer distributions; CG14 genome exhibited the smallest size of 399.8 Mbp, WAB56–104, NU3 and NU7 exhibited larger sizes ranging from 444.9 to 455.4 Mbp, and NU4 and NU5 genome sizes were intermediate between the parental lines (Table 1). To evaluate the reliability of those predictions, we analyzed publicly available sequencing data in cv. Nipponbare (temperate *japonica*), IR64 (indica), and an African variety Moroberekan (tropical *japonica*). The result of Nipponbare ranged from 371.1 to 421.3 Mbp, which was close to the actual genome size of 384.2 to 386.5 Mbp (Kawahara et al. 2013). The result of IR64 (355.6 Mbp) was smaller than that of Nipponbare, being consistent with the sizes of genome assemblies in both cultivars (Schatz et al. 2014). The result of Moroberekan was 446.8 Mbp, which was nearly identical to that of WAB56–104. These data supported the size diversity and the larger genome sizes of NU varieties and the parental variety compared to the Nipponbare genome.

**Table 1:** Prediction of genome size and sequencing depth

Variety	k-mer analysis		Flow cytometry	
	Genome size (Mb)	Sequencing depth (X)	DNA content (pg/2C)	Genome size (Mb)
WAB 56–104	446.982	38.9	0.896 ± 0.016	438.27 ± 7.86
CG 14	399.771	43.7	0.802 ± 0.006	392.06 ± 2.83
NERICA 3	444.887	32.5	0.849 ± 0.016	415.16 ± 7.65
NERICA 4	425.708	45.5	0.878 ± 0.012	429.46 ± 5.91
NERICA 5	424.061	53.8	0.845 ± 0.019	412.96 ± 9.48
NERICA 7	455.363	37.8	0.914 ± 0.024	446.70 ± 11.87
Nipponbare	371.142–421.300	–	0.863 ± 0.014	421.76 ± 6.71
IR64	355.615	–	–	–
Moroberekan	446.806	–	–	–

k-mer was set as “17”

It was notable that all the six varieties we sequenced exhibited larger genome sizes compared to Nipponbare. There are still controversial hypotheses on the origin of differences in genome size in different *O. sativa* genotypes. Burr (2002) reviewed the predicted size ranged from 403 to 430 Mbp, while Kawahara et al. (2013) mentioned the actual size of the sequenced Nipponbare genome was between 384.2 and 386.5 Mbp. Miyabayashi et al. (2007) observed that the DNA content of a wild rice *Oryza glumaepatula*, which has the AA genome, was 15.4% higher than that of the Nipponbare genome. To evaluate our *k*-mer-based estimation, nuclear DNA contents of the African varieties were determined by measuring PI-stained nuclei using flow cytometry. The estimated genome size by this way ranged from 392.1 Mb of CG14 to 446.7 Mb of NU7 (Table 1). The genome size of Nipponbare (421.8 Mb) was larger than those of the latest genome assembly, but it was close to the range given from the *k*-mer analysis. The DNA content of Nipponbare was smaller than those of NU7 and WAB 56–104 with statistical significance (Student’s *t*-test, at 10% level) and larger than that of CG14 with statistical significance (Student’s *t*-test, at 1% level). A smaller genome size of *O. glaberrima* than those of *O. sativa* was previously reported by Martínez et al. (1994) and Uozu et al. (1997). Remaining three varieties did not show any clear difference although their values were numerically different from that of Nipponbare. Taken together, one common observation which was not inconsistent with previous studies was that the recurrent parent WAB 56–104 had a larger genome than Nipponbare, and it is likely to be inherited in NU7. If our relative estimation of WAB56–104 and Nipponbare genome size is trustable, the WAB56–104 genome could have unique genetic information that was not found on the Nipponbare genome assembly. We examined this possibility by de novo assembly of unmapped sequence reads later (section “Unmapped sequences from WAB56-104”).

### Genomic comparison in the parental varieties

Parental SNPs, MNPs, and short InDels were mined using a bioinformatics pipeline. To consider genome sequence diversity between *O. sativa* and *O. glaberrima* (Wang et al. 2014), we set the mapping parameters after optimization. Against the Nipponbare reference genome, 3,088,818 SNPs, 103,568 MNPs, and 404,070 potential InDels were found (Table 2). Approx. 87.8% and 11.9% of the parental SNPs represented common nucleotide alleles between Nipponbare and WAB56–104 and Nipponbare and CG14, respectively. The MNPs were comprised of four categories: di, tri, tetra, and penta-nucleotide polymorphisms, and di-MNPs were the most abundant (97.0%; Additional file 3: Table S2). Majority (81.8%) of the MNPs represented the same nucleotide types as Nipponbare, but 6.2% were of CG14. For the short InDels, the length ranged from 1 to 45 bp, and 90% of these were 1–4 bp length (Additional file 4: Table S3). Most of the InDels (90.9%) were detected as changes in CG14 compared to the alleles of WAB56–104 and Nipponbare. These results are consistent with the knowledge that tropical *japonica* rice is genetically closer to temperate *japonica* than to *O. glaberrima*. More distant genetic relationship of WAB56–104 to *O. glaberrima* accessions was observed to Nipponbare (Semon et al. 2005). However, the actual similarity between Nipponbare and CG14 at polymorphic sites might be closer because a technical bias would exist when the sequence reads of the parental lines were aligned upon the Nipponbare genome sequence. Total of 42 polymorphic sites across all the chromosomes were tested by CAPS assays, and 40 of them were validated (Additional file 5: Table S4), suggesting more than 95% of mined polymorphisms in this study are genuine.

**Table 2:** Polymorphic sites between WAB 56–104 and CG 14

Chr	Nucleotide polymorphism		Insertion		Deletion		Total
	SNP	MNP	WAB56–104	CG 14	WAB56–104	CG 14	
1	360,381	11,386	1972	20,132	2804	24,478	49,386
2	316,206	10,336	1564	17,498	2320	21,101	42,483
3	306,733	9299	1293	16,964	1879	20,338	40,474
4	270,022	9601	1370	14,362	2061	17,316	35,109
5	251,323	8021	1377	13,075	2055	15,560	32,067
6	271,081	9447	1413	14,354	1997	17,557	35,321
7	254,203	8756	899	14,537	1314	17,184	33,934
8	221,116	7317	1131	11,605	1632	14,365	28,733
9	190,775	6350	666	10,347	893	12,284	24,190
10	201,301	7146	1771	9125	2600	11,457	24,953
11	238,964	8611	1884	11,917	2360	14,295	30,456
12	203,812	7230	1527	10,421	2084	12,631	26,663
Un	538	9	3	20	5	32	60
Sy	2363	59	7	113	2	119	241
Total	3,088,818	103,568	16,877	164,470	24,006	198,717	404,070

Hundred sites of structural variation between *O. sativa* and *O. glaberrima* were reported previously (Hurwitz et al. 2010). We searched potential structural variants, translocation breakends, inversions, tandem repeats and long InDels between WAB56–104 and CG14 *in silico*. By the reciprocal somatic calls, we predicted 8661 of translocation breakends, 329 of inversions, 320 of tandem duplications, and 4715 of long InDels (Table 3). The structrural variants were distributed across all the chromosomes (data not shown). The average length of structural variants was as follows: 1.5 Mbp for inversion, 59.4 kbp for tandem duplication, 101.3 kbp for long deletion, 1.7 kbp for long insertion. We also searched structural variations between Nipponbare and the two parental varieties separately. This computation revealed numerous structrual variants potentially existed between Nipponbare and the two parental varieties (Table 3). Number of structural variants in WAB56–104 ranged from 32.9% (long insertion) to 48.7% (tandem duplication) of those in CG14. Average length of inversions and tandem duplications in CG14 (1.54 Mbp and 65.5 kbp, respectively) was close to those in WAB56–104 (1.35 Mbp and 55.2 kbp, respectively), while average length of long InDels in WAB56–104 (1.8 kbp and 7.0 kbp, respectively) was longer than those in CG14 (1.4 kbp and 5.9 kbp, respectively). This observation is due to much more InDels, which are of relatively shorter length, found in CG14. Experimental validation of those structural variations remains to be performed.

**Table 3:** Structural variants in the parental lines and Nipponbare

Comparison	Interchromosomal translocation breakend	Inversion^*1^	Tandem duplication^*2^	Long deletion^*3^	Long insertion^*3^
WAB 56–104 vs. CG14	6807	283	244	3741	584
CG14 vs. WAB56–104	1238	46	76	336	54
Nippobare vs. WAB 56–104	3346	165	154	2222	282
Nippobare vs. CG14	9176	381	316	6071	857

*1 Inversion between 100 bp to 10 Mbp

*2 Tandem duplication between 100 bp to 1 Mbp

*3 Insertion and deletion between 50 bp to 1 Mbp

### Polymorphisms in NU varieties

**Fig. 1:**
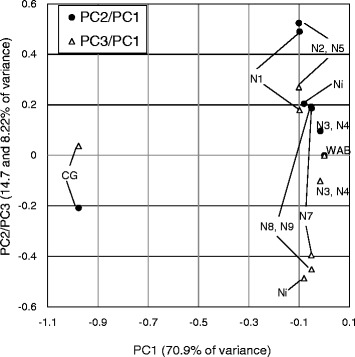
PCA score plot for representing genotypic diversity. The X-axis represents PC1 and the Y-axis shows PC2 and PC3. Ni: Nipponbare, WAB: WAB56–104, CG: CG14, N1 to N9: NERICA 1 to NERICA 9

For polymorphisms among NU varieties, the new sequencing data of the four NU genomes 3, 4, 5 and 7 were used along with the publicly available sequences of the other four NU genomes 1, 2, 8, and 9 (Additional file 6: Table S5). Polymorphisms between each NERICA and both parents were called, and a total of 4,461,719 polymorphic sites were found for SNPs, MNPs and short InDels (Table 4). Population structure of the 10 varieties (8 NUs and 2 parents) was analyzed using common polymorphic sites (1,026,367 sites). Principal component analysis (PCA) was used to dissect variance of allelic divergence of the 10 genomes (Fig. 1). PC1, which explained 70.9% variance, represented a large part of the difference between the two parental genomes. PC2 and PC3, which explained 14.7% and 8.22% variance, respectively, represented differences among the NU varieties. NU3 was plotted at the same location as NU4, and at a different site NU8 and NU9 were plotted at the same location. This could most likely be due to NU3 being a sister line of NU4 and NU8 being a sister line of NU9.

**Table 4:** Number of polymorphisms in the upland NERICA

Genotype	Category	Allele type			CG 14 allele (%)		Non-parental allele (%)	
		WAB56–104	CG 14	Non-parental	Category	All	Category	All
NERICA 1	SNP	2464390	247641	101458	8.80	9.03	3.61	3.81
	MNP	79607	6336	8975	6.68		9.46	
	Short InDel	324348	43036	14852	11.26		3.89	
NERICA 2	SNP	1485308	148296	42301	8.85	8.68	2.52	2.65
	MNP	46795	3990	3566	7.34		6.56	
	Short InDel	171010	14367	5123	7.54		2.69	
NERICA 3	SNP	2989656	41101	39623	1.34	1.32	1.29	1.39
	MNP	98929	671	3991	0.65		3.85	
	Short InDel	333122	4601	5371	1.34		1.57	
NERICA 4	SNP	3008891	41574	40255	1.35	1.33	1.30	1.41
	MNP	99336	681	4059	0.65		3.90	
	Short InDel	322610	4520	5457	1.36		1.64	
NERICA 5	SNP	2637694	293878	79951	9.76	9.81	2.65	2.91
	MNP	85743	8884	7838	8.67		7.65	
	Short InDel	275398	34231	12349	10.63		3.84	
NERICA 7	SNP	2471034	117058	82482	4.38	4.32	3.09	3.36
	MNP	74904	1816	8946	2.12		10.44	
	Short InDel	272139	12842	11127	4.34		3.76	
NERICA 8	SNP	1476391	98444	96093	5.89	5.82	5.75	6.05
	MNP	46196	1787	8550	3.16		15.12	
	Short InDel	164605	11231	11142	6.01		5.96	
NERICA 9	SNP	1786210	120441	116935	5.95	5.88	5.78	6.10
	MNP	54940	2150	10599	3.18		15.66	
	Short InDel	203351	13928	14197	6.02		6.13

The number of candidate polymorphisms discovered revealed that the NU varieties contained the donor alleles in different proportions, which ranged from 1.32 to 9.03% (Table 4). For example, NU5 exhibited the highest proportion of the CG14-specific SNPs (9.81%). Comparatively lower proportions of the CG14-specific SNPs at 1.32 and 1.33% were noted in NU3 and NU4 respectively. These results reflected different amount of introgression segments from CG14. Frequency of the non-parental SNPs among the total SNPs ranged from 1.39 to 6.10%. These numbers were compatible with the frequency of non-parental loci of 2.2% obtained by SSR maker-mediated genotyping (Semagn et al. 2007). Non-parental allele polymorphisms were the most frequent in NU9 and the least frequent in NU3 and NU4 (Table 4). Notably, the rates of CG14 allele and non-parental allele were at the same level, which implied that mutations, chromosomal rearrangements, or perhaps out-crossing contributed to the diversity of the NU genomes.

### Chromosomal structure in NU varieties

**Fig. 2:**
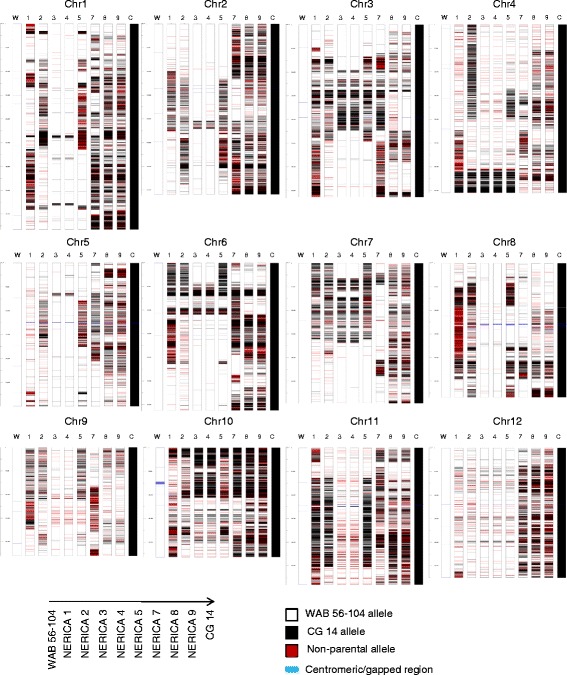
Graphical genotype of the upland NERICA and the parental lines. W: WAB56–104, 1 to 9: NERICA 1 to NERICA 9 and, C: CG14. Introgression segments from CG14 are colored in black. Non-parental segments are represented in red. Centrometic and gap regions are shown in light blue. Scale bar is standardized as 100 bp

The unique breeding processes of NU varieties could bring genomic alterations in various ways: de novo mutations, chromosomal rearrangement, translocation, chromosomal loss, etc., which might be required for stabilization of hybrid genomes and adaptive evolution (Baack and Rieseberg 2007; Rieseberg and Willis 2007; Morales and Dujon 2012). The chromosomal structure of the NU varieties was inferred using genetic imputation, by considering all polymorphic sites to predict the recipient parent segments of WAB56–104, the introgression segments from CG14, and potential non-parental segments. For the proportion of CG14 segments among NU varieties a wide range of 1.40 in NU3 and 4 to 10.1% in NU2 was noted. Similarly, the potential non-parental segments ranged from 0.090 of NU3 and 4 to 2.74% of NU9. Graphical genotype revealed the chromosomal structures of the NU varieties (Fig. 2). Reiterating the result from PCA, NU2 and 5, NU3 and 4 and NU8 and 9 exhibited highly related genotypes, while NU2 and 5 revealed large differences in introgressions from CG14 on Chr 4. NU1 and 5 exhibited similarities at limited regions for example at the periphery of the centromeric region on the Chr 2, 3, 7 and 11, the upper edge of Chr 6, 7, and 9, and the bottom edge of Chr 4. These results suggested that the known NU varieties originated from a limited number of independent interspecific hybridization events but that sister lines were identified as independent events, adding to the number of apparently independent NU varieties. Frequent distribution of short non-parental segments could be due to a combination of factors such as structural variants between Nipponbare and the parental lines, de novo mutations and genomic alterations at the chromosomal level. However, some large non-parental segments were observed. For example, in NU1 the middle part of Chr 8, which contained a fragrance gene *BADH2* from WAB638–1 (Asante et al. 2010). Chr 1 in NU5 and Chr 7 in NU7 also indicated non-parental segments. In the previous genotyping by microsatellite markers (Semagn et al. 2007), those non-parental segments were not detected.

Common introgression segments from CG14 across the eight NU varieties totaled 637,849 bp, composed from 3168 segments. These were frequent in Chr 6 (1608 segments) and 10 (1143 segments) (Additional file 7: Table S6). Also, the common introgression segments were closely distributed (within 1.4 to 1.8 Mbp) on each of these two chromosomes. For example on Chr 6, they occurred from 5,613,148 to 7,010,047 and on Chr 10 from 174,587 to 1,974,978. These fragmented segments might be due to structural variations between NERICA and Nipponbare/WAB56–104 or due to recombination between unlinked genes. The longest introgression segment was 24,125 bp on Chr 7 (3,885,715–3,909,839), which was followed by 23,541 bp on Chr 10 (1,501,250–1,524,790). To examine gene composition on the common introgression segments from CG14, enrichment of GO terms were tested. It appeared that 11 GO terms, including ‘cell wall biogenesis’ [GO:0042546] and ‘transferase activity, transferring glycosyl groups’ [GO:0016757], were over-represented. That enrichment was due to five xyloglucan fucosyltransferase genes that were located at the same locus (Chr 6: 5,701,639–5,734,237) (Additional file 8: Table S7). Although this enrichment alone did not look biologically informative, this locus was co-localized with quantitative trait loci (QTLs) of typical agronomic traits and grain quality between *O. sativa* and *O. glaberrima* (Lorieux et al. 2000; Aluko et al. 2004). The facts might imply that the locus containing the xyloglucan fucosyltransferase genes was positively acted on during the selection process for developing the NU varieties. Since the region of Chr6: 5,613,148 to 7,010,047 contains 207 genes in the Nipponbare genome, we could not specify genes relevant to the QTLs in this study.

Xyloglucan fucosyltransferase catalyzes transfer of Fucose (Fuc) from GDP-Fuc to a galactosyl residue of xyloglucan in the process of fucogalactoxyloglucan biosynthesis. Xyloglucan is a key component in cell walls and can play a role in cell wall structure and function. Recently, fucogalactoxyloglucan, which is widely distributed across dicot plants, was surprisingly detected in root hairs and anthers in rice (Liu et al. 2015), however, the biological function of fucogalactoxyloglucan is still unclear. The five xyloglucan fucosyltransferase genes were categorized into three groups based on their protein sequence similarities (Additional file 9: Figure S2). *LOC_Os06g10910* is a candidate fucosyltransferase coexpressed with a xyloglucan glycosyltransferase *OsCSLC3* (Liu et al. 2015). *LOC_Os06g10920* was a probable ortholog of *AtFUT3*, of which overexpression altered cell wall composition and detrimental (Sarría et al. 2001). We observed several CG14-alleles that were introgressed into NU varieties caused amino acid substitutions in these genes (Additional file 10: Figure S3). Many CG14-alleles were also observed in the promoter regions. Cell wall in plants is the primary component that can determine the physical properties and protects them from against biotic and *a*biotic stress (Zabotina 2012). These genes from CG14 might affect on agronomic characteristics in the upland NU varieties.

Variety-specific/lineage-specific introgression segments were also predicted in all the NU varieties (Additional file 11: Table S8). The total nucleotide ranged from 81.9 kbp in NU4 to 6.85 Mbp in NU8 and 9 lineage. Number of genes with the segments varied from 218 in NU4 to 4906 in NU8 and 9 lineage. Various kinds of genes that would be from CG14 were found (Additional file 12: Table S9). Gene Ontology enrichment analysis (GOEA) indicated 109 over-represented GO terms, implying biased distribution of particular genes in NU1, 2, 5, 7 and the two lineage of NU2 and NU5, and NU8 and NU 9 (Additional file 13: Table S10). The listed GO categories might relate to their variety-specific characteristics, however, the biological meaning remains to be examined.

### Gene composition relevant to traits

**Fig. 3:**
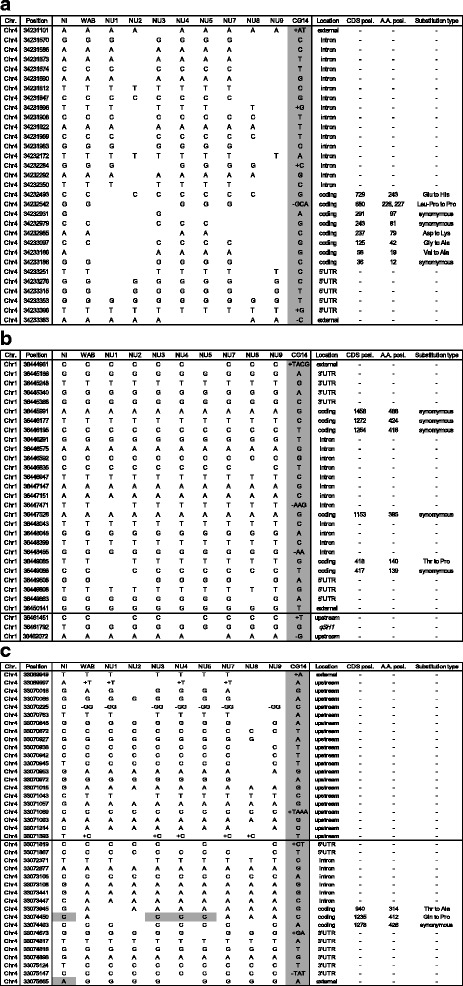
Graphical representation of allelic patterns in the three grain shattering genes. Substitution type indicates amino acids of WAB56–104-allele to CG14-allele. **a** SHATTERING 3, **b** qSH1, and **c** SHAT1. Varietal names are abbreviated as follows: Nipponbare, Ni; WAB56–104, WAB; NERICA 1, NU1; NERICA 2, NU2; NERICA 3, NU3; NERICA 4, NU4; NERICA 5, NU5; NERICA 7, NU7; NERICA 8, NU8; NERICA 9, NU9. CDS posi. and A.A. posi. Represent positions in coding sequences and positions in deduced amino acid sequences, respectively. Alleles of CG14 are colored in gray

Grain shattering habit is the major constraint in *O. glaberrima* rice production (Africa Rice Center 2008). In agreement with disappearance of this trait in the NU varieties, key genes for grain shattering, *SHATTERING 3/SEED SHATTERING 4* (LOC_Os04g57530, Li et al. 2006), *qSH1* (LOC_Os01g62920, Konishi et al. 2006), and *SHAT1* (LOC_Os04g55560, Zhou et al. 2012), were on chromosomal segments of WAB56–104 (Fig. 3). In the case of *SHATTERING 3*, five of non-synonymous parental polymorphisms were on the coding region, and one of the polymorphisms was the identical allele associated with shattering habits (Asp for non-shattering, Lys for shattering) (Fix. 3a). The causal allele of *qSH1* was not polymorphic among the African varieties, however, one non-synonymous SNP was found in CG14 (Fix. 3b). This SNP locates on the homeobox KN domain [PF05920] and substitutes Thr of WAB 56–104 into Pro of CG14. The structural and biochemical properties of the two amino acids might affect the function of the *qSH1* gene. *SHAT1* exhibited a mixed allelic pattern in the genic region while the probable promoter region of NU varieties consistently showed only of WAB 56–104 alleles (Fig. 3c). The corresponding CG14 segments, which are responsible for shattering, would be excluded in the processes of backcrossing followed by the artificial selection.

Discovery of trait-associated alleles helps in elucidating the genetic reasons underlying target trait variation. The high-resolution graphical genotype is useful to check possibility of association with trait among the NU varieties and the parental varieties when genes, for which function was determined, showed allelic divergence. For example, Kishine et al. (2008) reported that the allele of *granule-bound starch synthase I* (*GBSSI*) from CG14 was associated with high grain amylose content in NU1, 2 and 5.Our graphical genotype indicated association of this gene with the corresponding CG14 introgression segment (Table 5, Additional file 14: Figure S4a). Also, a fragrant rice allele *BADH2*, which was found in NU1 (Asante et al. 2010), was located on the three non-parental segments of Chr8: 20379746–20,399,750 in NU1 genotype uniquely as expected (Table 5). Ikeda et al. (2009) documented the awnness in NU2 and 5, divergent apiculous color in NU1, 2 and 5, and distinct stigma color in NU1. It appeared that NU2 and 5 conserved CG14 alleles on *Regulator of Awn Elongation 1* (*RAEI*), which is one of genes involved in awn formation in CG14 (Furuta et al. 2015) (Table 5). We noted that *OsC1*, which is a determinant of anthocyanin accumulation in rice (Chin et al. 2016), was associated with apiculous color among the 8 NU varieties and the parental varieties (Table 5). Although these ‘gene-trait relationships’ need extensive analyses using other NU varieties or a rice diversity panel, the graphical genotype we have presented can be a starting point and also guides functional validation by molecular genetics.

**Table 5:** Key genes segregating among the NU varieties

Gene abbreviation	Locus	Trait	Reference	Causal origin	Variety with the gene segment
*GBSSI*	LOC_Os06g04200	Grain amylose content	Kishine et al. (2008)	CG14	NU1, 2, and 5
*BADH2*	LOC_Os08g32870	Grain fragrance	Asante et al. (2010)	WAB638–1 (outcross)	NU1
*RAE1*	LOC_Os04g28280	Awnness	Furuta et al. (2015)	CG14	NU2 and 5
*OsC1*	LOC_Os06g10350	Purple pigment	Chin et al. (2016)	CG14	NU1, 2, and 5
*OsSWEET14*	LOC_Os11g31190	Bacterial leaf blight susceptibility	Hutin et al. (2015)	CG14	NU1, 2, and 5
*OsHKT1;5*	LOC_Os01g20160	Salt tolerance	Platten et al. (2013)	WAB56–104	NU3 and NU4

Gene sequence information of the NU varieties is useful to explore divergent traits and their origin. Here we introduce one example each for biotic and *a*biotic stress tolerance (Table 5). *OsSWEET14* is a susceptibility gene to bacterial leaf blight in wild rice (Hutin et al. 2015). The corresponding locus in the graphical genotype showed that NU1, 2, and 5 had the CG14 alleles (Additional file 14: Figure S4b). Association analysis of those alleles and resistance to bacterial blight may lead to discover differences of NU varieties. Notably, Séré et al. (2005) reported the different susceptibility of NU1 (with *OsSWEET14* from CG14) and NU4 (without *OsSWEET14* from CG14).One of salt tolerance determinant gene *OsHKT1;5* (Platten et al. 2013), which encodes a Na^+^ transporter, exhibited variation in the NU varieties. Namely, NU3 and NU4 were identical to WAB56–104, but other NU varieties contained non-parental segments in this gene region. This observation is consistent with the concept that salt tolerance in NU4 is derived from WAB56–104 as reported previously (Awala et al. 2010). Interestingly, Yamamoto et al. (2011) reported the similar physiological response of NU3 and NU4 to salt stress compared to other five NU varieties, while NU3 and NU4exhibited different profiles of total amino acids and polyamine. At the same time, Yamamoto et al. (2011) reported salt stress susceptibility of NU2 among the NU varieties. Those differences of NU varieties in salt stress response imply that multiple genetic components for salt stress adaptation are combined differently in their genomes. Platten et al. (2013) reported some *O. glaberrima* seem to have another salt exclusion mechanism that is independent on *OsHKT1;5*. Other seven Na^+^ transporter genes (Platten et al. 2006) exhibited no difference in the chromosomal segments in NU3 and NU4 (Additional file 15: Table S11). Further investigation is required to specify the reason.

### Unmapped sequences from WAB 56–104

More than 10% of the NU sequences were not mapped correctly on the rice reference genome of Nipponbare. To characterize those sequences, we performed de novo assembly of unmapped reads from WAB56–104. Total of 37,894 genomic scaffolds were constructed; those covered approx. 19 Mb of genomic scaffolds (Additional file 16: Table S12). The smaller value of total nucleotide of the genomics scaffolds than expected would be due to duplication of DNA segments. As evidence, a computational survey predicted 578 of chromosomal regions exhibiting higher copy number variation in WAB56–104 upon the Nipponbare genome (with E-value of 0.01), and notably, it was more than 460 sites in CG14. Hence, we assumed that some unmapped WAB56–104 sequence reads were mapped on highly related paralogous chromosomal regions in Nipponbare.

Structural annotation of long assembled genomic scaffolds (no less than 1 kb) by Augustus (Stanke and Morgenstern 2005) predicted 653 genes. A large part of the predicted gene proteins had homology with cereal proteins (Additional file 16: Table S12 and Additional file 17: Table S13). Notably, 95.6% (624) did not show strong sequence homology (less than 90% identity) with Nipponbare proteome sequences (Additional file 17: Table S13). Genes with particular functional annotations showed frequent localization on the scaffolds with statistical significance (in Fisher’s exact test at 5% level); 35 genes for disease resistance protein (*p* value = 0), 10 genes for wall-associated receptor kinase (WAK) (*p* value = 0), seven genes for leucine-rich repeat (LRR) receptor-like serine/threonine-protein kinase (*p* value = 5.6E^− 3^), six genes for lectin receptor kinase (LRK) (*p* value = 0), and five genes for cytochrome P450 (*p* value = 4.4E^− 2^). Potential *a*biotic stress-related genes (25) were obviously observed prominently. WAKs, LRR receptor-like serine/threonine-protein kinases, LRKs can be involved in pathogen resistance and *a*biotic stress response (Afzal et al. 2008; Kanneganti and Gupta 2008; Vaid et al. 2013). A WAK was implicated as one of the NAM transcription factor regulated genes in drought tolerance (Dixit et al. 2015). Those maternal genes might be the basis for the characteristics in WAB56–104, perhaps for biotic and *a*biotic stress tolerance. Sakai et al. (2011) observed more frequent distribution of proteins with protein kinase domains, leucine-rich repeat domains, and disease resistance gene on unmapped sequences from *O. glaberrima*.

To analyze whether the maternal genes above exist in the NU genomes or not, we re-mapped unmapped sequence reads of NU3, 4, 5, and 7 upon the genomic scaffolds of unmapped WAB56–104 sequences. The result indicated that 56 genes exhibited segregation among the four NU varieties and CG14 (Additional file 17: Table S13). Fifty one of them were segregated among the four NU varieties. Total of 21 genes were functionally annotated by a BLAST search against the Swiss-Prot database (Table 6). Most of the genes exhibited WAB56–104 allele in NU varieties except two. It is unknown how those genes might contribute on divergent agronomic traits related to biotic and *a*biotic stress resistance in the NU and parental varieties. Association analysis using DNA markers of those genes may lead to discovery of useful genes in agronomy.

**Table 6:** Occurrence of WAB 56–104 specific genes in NU varieties and CG 14

Category	Gene identifier	Functional annotation		Gene occurrence					
		Gene of BLAST hit in Swiss prot	Species of BLAST hit in Swiss-prot	NU3	NU4	NU5	NU7	CG14	Remarks
Disease	g13.t1	Putative disease resistance protein RGA4	*Solanum bulbocastanum*	No	No	No	Yes	No	WAB 56–104 allele
	g51.t1	Putative disease resistance RPP13-like protein 3	*Arabidopsis thaliana*	Yes	Yes	Yes	No	No	WAB 56–104 allele
	g98.t1	Disease resistance protein RPP13	*Arabidopsis thaliana*	Yes	Yes	No	Yes	Yes	WAB 56–104 allele
	g108.t1	Putative disease resistance RPP13-like protein 1	*Arabidopsis thaliana*	No	No	No	No	Yes	Polymorphic in parents
	g342.t1	Chitin elicitor-binding protein	*Oryza sativa*	Yes	Yes	No	No	No	WAB 56–104 allele
	g632.t1	Putative disease resistance RPP13-like protein 2	*Arabidopsis thaliana*	Yes	Yes	No	Yes	No	WAB 56–104 allele
Receptor	g85.t1	Wall-associated receptor kinase-like 18	*Arabidopsis thaliana*	Yes	Yes	Yes	No	Yes	Both parental allele
	g115.t1	Wall-associated receptor kinase-like 18	*Arabidopsis thaliana*	Yes	Yes	Yes	No	Yes	Both parental allele
	g227.t1	Wall-associated receptor kinase 2	*Arabidopsis thaliana*	Yes	Yes	No	Yes	No	WAB 56–104 allele
	g162.t1	Receptor-like protein 12	*Arabidopsis thaliana*	Yes	Yes	Yes	No	Yes	WAB 56–104 allele in NU3 and NU4.CG 14 allele in NU5.
	g418.t1	G-type lectin S-receptor-like serine/threonine-protein kinase LECRK3	*Oryza sativa*	Yes	Yes	No	No	No	WAB 56–104 allele
Others	g173.t1	11-oxo-beta-amyrin 30-oxidase	*Glycyrrhiza uralensis*	No	No	Yes	No	Yes	Unspecified
	g268.t1	Uncharacterized protein	*Arabidopsis thaliana*	Yes	Yes	No	Yes	No	WAB 56–104 allele in NU3 and NU4. Unspecified in NU7.
	g338.t1	F-box/FBD/LRR-repeat protein	*Arabidopsis thaliana*	No	No	No	No	Yes	Polymorphic in parents
	g361.t1	Cytochrome P450 87A3	*Oryza sativa*	Yes	Yes	No	Yes	No	Both parental allele
	g427.t1	UPF0481 protein	*Arabidopsis thaliana*	Yes	Yes	No	No	No	WAB 56–104 allele
	g453.t1	Tetraspanin-8	*Arabidopsis thaliana*	Yes	Yes	No	No	No	WAB 56–104 allele
	g500.t1	Protein Brevis radix-like 2	*Arabidopsis thaliana*	Yes	Yes	Yes	No	Yes	WAB 56–104 allele
	g517.t1	UDP-glycosyltransferase 76E11	*Arabidopsis thaliana*	Yes	Yes	Yes	No	Yes	WAB 56–104 allele
	g597.t1	Basic blue protein	*Cucumis sativus*	Yes	Yes	Yes	No	Yes	WAB 56–104 allele
	g643.t1	Autonomous transposable element EN-1 mosaic protein	*Zea mays*	No	No	Yes	No	No	WAB 56–104 allele

## Conclusions

Interspecific hybrids NU varieties have a great potential for improvement of rice, especially for Africa. Due to the unique breeding processes, the NU genomes required in-depth analyses. The present study provided a draft whole picture of the NU genomes, including polymorphisms, introgression from the donor parent, some potential introgression segments by outcross and de novo mutations.

Currently, short length polymorphisms, especially SNPs have played a central role in rice breeding, genetics, and biology (Feltus et al. 2004). SNP is the ultimate DNA marker which can be detected by several methods such as SNP chip, next generation sequencing, restriction enzymes, and real-time PCR (Kim and Misra, 2007; Varshney et al. 2009). SNP genotyping data are useful for QTL mapping for agronomic traits, genome wide association study, and measurement of genetic distance (Yonemaru et al. 2010; Korte and Farlow 2013). Since SNPs can be relevant in terms of biochemical function of protein and gene regulation, nucleotide substitutions themselves could be causal of particular traits and thus be markers for breeding (Anderssen and Lübberstedt 2003; Gupta and Rustgi 2004). Approx. 81% of our polymorphic sites were on genic regions, and 43% were on promoter regions. In the NU varieties, divergence was observed in quantitative agronomic traits and apparent phenotypes (Ikeda et al. 2009; Sanni et al. 2009; Fukuta et al. 2012; Saito et al. 2012; Rodenburg et al. 2015). Studies over the past years have also accumulated data that captures the physiological behavior of the NUs and their divergence in *a*biotic stress responses (Yamamoto et al. 2011; Atayese et al., 2016; Sikuku et al. 2012; Sakariyawo et al. 2015). In most cases the genetic reasons for divergence in traits are not known. The polymorphism panel resource we created is useful for identification of agronomically useful traits in rice.

In conclusion, our analyses revealed genome characteristics of the NU varieties and the parental varieties. The established whole genome polymorphic resource and knowledge are useful for addressing the genetic reasons of the prominent agronomic characteristics in the NERICA and promotes marker-assisted selection in the development of new rice cultivars. This is especially useful in the light of the critical information about the close relationship between pairs of some NERICA varieties. The genotyping matrix for the 8 NU and the parental lines is available upon request.

## Additional files

Additional file 1: **Table S1.** Summary of whole genome sequencing data. (XLSX 12 kb)

Additional file 2: **Figure S1.** *k*-mer analysis (*k*-merlength = 17). The X-axis shows *k*-mer depth, and the Y-axis shows proportion that represents the frequency at that *k*-mer depth divided by to total frequency of all *k*-mer depth. (a) WAB56–104, (b) CG14, (c) NERICA 3, (d) NERICA 4, (e) NERICA 5, (f) NERICA 7. Arrow indicates a peak of heterogeneous sequences.Peak between 0 to 8 of *k*-mer depth would be due to sequencing error or contaminants. (PPTX 109 kb)

Additional file 3: **Table S2.** MNPs between WAB 56–104 and CG 14. (XLSX 9 kb)

Additional file 4: **Table S3.** Distribution of InDel length. (XLSX 12 kb)

Additional file 5: **Table S4.** Validation result of parental polymorphic sites. (XLSX 12 kb)

Additional file 6: **Table S5.** Public whole genome sequence data in upland NERICA. (XLSX 9 kb)

Additional file 7: **Table S6.** Common introgression segments from CG 14. (XLSX 104 kb)

Additional file 8: **Table S7.** Location of xyloglucan fucosyltransferase genes on common long introgression segments from CG 14. (XLSX 11 kb)

Additional file 9: **Figure S2.** Phylogeny of genes for fucogalactoxyloglucan biosynthesis**.** Predicted proteins sequences were aligned by ‘CLUSTAL Ω’ and a phylogenetic dendrogram was constructed by ‘Simple Phylogeny’ using the neighbor-joining method with the option of ‘exclude gaps’. Twelve fucosyltransferase genes in Arabidopsis (AtFUT1 to AtFUT12), four candidate genes for galactosyltransferase in rice, four candidate genes for acetyltransferase genes in rice were analyzed together with the five fucosyltransferase genes with CG14-alleles. The five fucosyltransferase genes of interest were marked with a circle. (PPTX 85 kb)

Additional file 10: **Figure S3.** Graphical representation of allelic patterns in the two fucosyltransferase. Substitution type indicates amino acids of WAB56–104-allele to CG14-allele. (a) LOC_Os06g10910 and (b) LOC_Os06g10920. Varietal names are abbreviated as follows: Nipponbare, Ni; WAB56–104, WAB; NERICA 1, NU1; NERICA 2, NU2; NERICA 3, NU3; NERICA 4, NU4; NERICA 5, NU5; NERICA 7, NU7; NERICA 8, NU8; NERICA 9, NU9. CDS posi. and A.A. posi. Represent positions in coding sequences and positions in deduced amino acid sequences, respectively. Alleles of CG14 are colored in gray. (PPTX 205 kb)

Additional file 11: **Table S8.** Variety/lineage-specific introgression segments from CG 14. (XLSX 11 kb)

Additional file 12: **Table S9.** Genes on variety/lineage-specific introgression segments from CG 14. (XLSX 1971 kb)

Additional file 13: **Table S10.** Enriched Gene Ontology on the introgression segments from CG 14. (XLSX 20 kb)

Additional file 14: **Figure S4.** Graphical genotype of *GBSSI* and *OsSWEET14* at gene level. Polymorphic position is indicated by vertical line. WAB56–104 segment is presented by while rectangle, and CG14 allele is presented in gray rectangle. Arrow represent the position of gene. (a) *GBSSI*, (b) OsSWEET14. Representative polymorphic sites were used. (PPTX 76 kb)

Additional file 15: **Table S11.** Allelic patterns of LOC_Os01g20160 (OsHKT1;5). (XLSX 28 kb)

Additional file 16: **Table S12.** Summary of genome assembly and annotation of unmapped reads from WAB 56–104. (XLSX 11 kb)

Additional file 17: **Table S13.** Genes on the genome assembly of unmapped sequences from WAB 56–104. (XLSX 180 kb)

## Abbreviations

CAPS: Cleaved amplified polymorphic sequences
Fuc: Fucose
GBSSI: Granule-bound starch synthase I
GO: Gene ontology
GOEA: Gene ontology enrichment analysis
InDel: Insertion and deletion
LRK: Lectin receptor kinase
LRR: Leucine-rich repeat
MNP: Multi nucleotide polymorphism
NERICA: New Rice for Africa
NU: Upland New Rice for Africa
PCA: Principal component analysis
PI: Propidium iodide
QTL: Quantitative trait locus
RAE1: Regulator of Awn Elongation 1
SNP: Single nucleotide polymorphism
SSR: Simple sequence repeat
WAK: Wall-associated receptor kinase
WARDA: The West Africa Rice Development Association
