# Supplementary material for: Comparative whole genome re-sequencing analysis in upland New Rice for Africa: insights into the breeding history and respective genome compositions
Source: Rice (N Y). 2018 May 15;11:33. doi: 10.1186/s12284-018-0224-3 (PMC5953909; doi:10.1186/s12284-018-0224-3)
Supplement: Supplementary file 2 — Figure S1. k-mer analysis (k-merlength = 17). The X-axis shows k-mer depth, and the Y-axis shows proportion that represents the frequency at that k-mer depth divided by to total frequency of all k-mer depth. (a) WAB56–104, (b) CG14, (c) NERICA 3, (d) NERICA 4, (e) NERICA 5, (f) NERICA 7. Arrow indicates a peak of heterogeneous sequences.Peak between 0 to 8 of k-mer depth would be due to sequencing error or contaminants. (PPTX 109 kb) [file 12284_2018_224_MOESM2_ESM.pptx]

## Slide 1
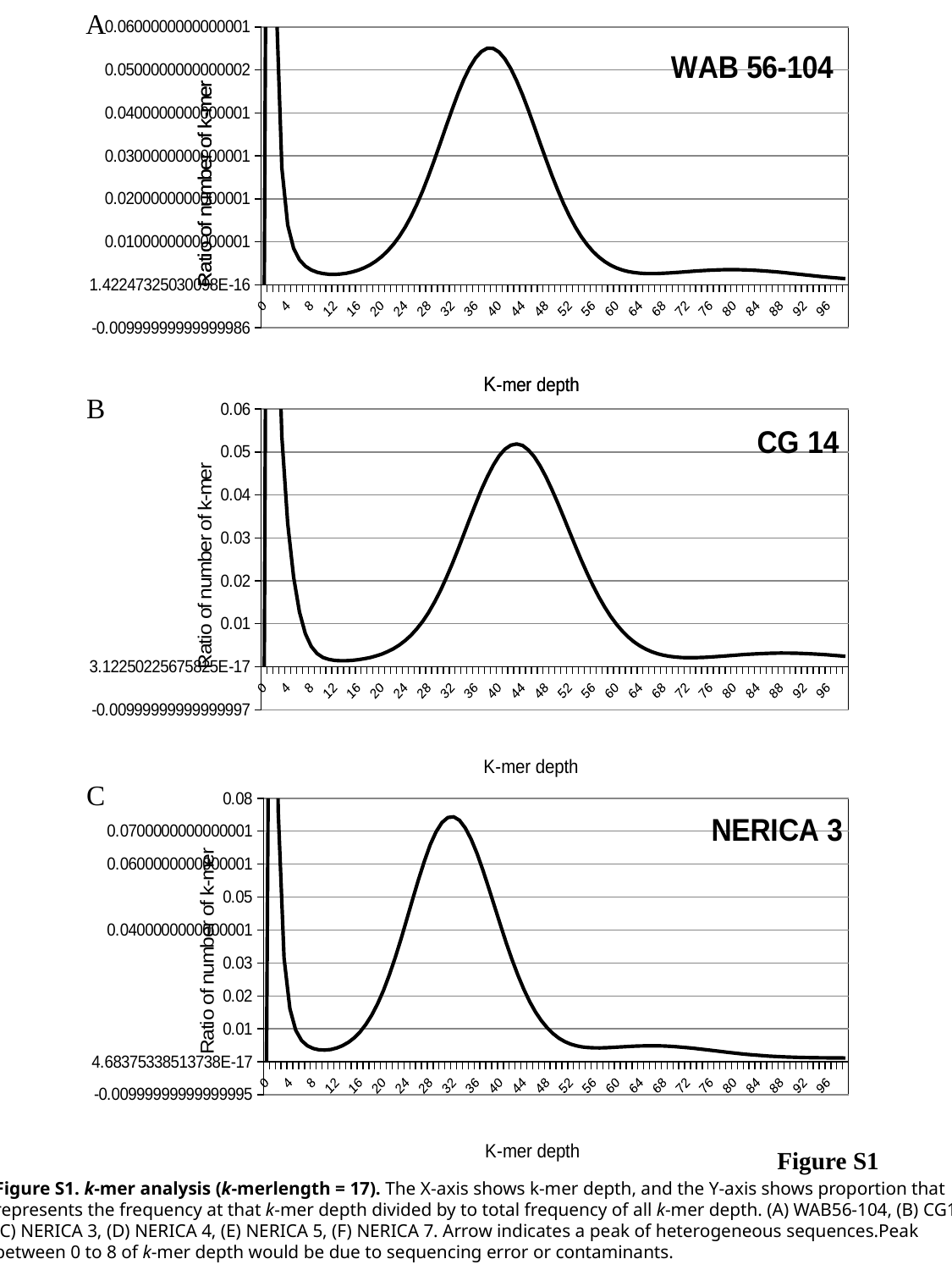

A
### Chart:
| Category | WAB 56-104 |
|---|---|
| 0 | 0.0 |
| 1 | 0.2740041368072564 |
| 2 | 0.06793562865360053 |
| 3 | 0.02718578033132175 |
| 4 | 0.0139275742624207 |
| 5 | 0.008470279186515571 |
| 6 | 0.00581090870070669 |
| 7 | 0.0043260133085727724 |
| 8 | 0.0034423386679872173 |
| 9 | 0.0028947401690204142 |
| 10 | 0.002592483871353504 |
| 11 | 0.0024327524288831667 |
| 12 | 0.002403719165409662 |
| 13 | 0.002494082274106261 |
| 14 | 0.002668869480441125 |
| 15 | 0.0029749025555534086 |
| 16 | 0.003385983671604544 |
| 17 | 0.003916780645531152 |
| 18 | 0.0046049585599530155 |
| 19 | 0.0054828254068235065 |
| 20 | 0.006553382069732119 |
| 21 | 0.007851150120162098 |
| 22 | 0.009391265193404321 |
| 23 | 0.011236249500975024 |
| 24 | 0.013375189182975524 |
| 25 | 0.015877058921149607 |
| 26 | 0.01871901548756505 |
| 27 | 0.021901635097511692 |
| 28 | 0.02539032771828383 |
| 29 | 0.029106117624539688 |
| 30 | 0.03301447753969408 |
| 31 | 0.03695091796630799 |
| 32 | 0.04079409709246953 |
| 33 | 0.04450285831320763 |
| 34 | 0.04781283291244902 |
| 35 | 0.05058737228993381 |
| 36 | 0.052813794004181056 |
| 37 | 0.05429863805039795 |
| 38 | 0.055074315112371874 |
| 39 | 0.055012814114092555 |
| 40 | 0.05416459668169869 |
| 41 | 0.05256909077429172 |
| 42 | 0.050309145881390495 |
| 43 | 0.04747400120662148 |
| 44 | 0.04420715717760222 |
| 45 | 0.04059214789607812 |
| 46 | 0.0368214349740118 |
| 47 | 0.03294766509156325 |
| 48 | 0.029127990985152192 |
| 49 | 0.02544847411665715 |
| 50 | 0.022010756581193815 |
| 51 | 0.01881730868787654 |
| 52 | 0.015944590212117676 |
| 53 | 0.01339509428343909 |
| 54 | 0.011203667664131422 |
| 55 | 0.009330243423682803 |
| 56 | 0.007742182674230842 |
| 57 | 0.006435155243447642 |
| 58 | 0.005386717260532511 |
| 59 | 0.004549459328852548 |
| 60 | 0.003899214636838878 |
| 61 | 0.0034186739053839203 |
| 62 | 0.003066121429532197 |
| 63 | 0.0028236260740710424 |
| 64 | 0.0026731882425652218 |
| 65 | 0.0025998605680859262 |
| 66 | 0.0025799668778261208 |
| 67 | 0.0026004938343947375 |
| 68 | 0.002663415403042686 |
| 69 | 0.0027377186499436654 |
| 70 | 0.0028328055829979193 |
| 71 | 0.0029350124831999265 |
| 72 | 0.0030338647834957898 |
| 73 | 0.0031409552509081284 |
| 74 | 0.0032269653668693683 |
| 75 | 0.003314487334829117 |
| 76 | 0.003385430276722071 |
| 77 | 0.00344079258537741 |
| 78 | 0.0034798839434675055 |
| 79 | 0.003503058067309011 |
| 80 | 0.003514080324143521 |
| 81 | 0.0034901417166499395 |
| 82 | 0.003454422073771624 |
| 83 | 0.003396435418250932 |
| 84 | 0.0033200726295708285 |
| 85 | 0.0032272620321671915 |
| 86 | 0.003130817284865204 |
| 87 | 0.0030178562676171 |
| 88 | 0.002887603086567021 |
| 89 | 0.00274629341807114 |
| 90 | 0.002602091262921491 |
| 91 | 0.0024588190393784546 |
| 92 | 0.0023136184913995837 |
| 93 | 0.0021684179434207237 |
| 94 | 0.00202479200356107 |
| 95 | 0.0018850911737956907 |
| 96 | 0.00175399934276907 |
| 97 | 0.0016269239034668345 |
| 98 | 0.001511806357707618 |
| 99 | 0.001406239152497568 |B
### Chart:
| Category | CG 14 |
|---|---|
| 0 | 0.0 |
| 1 | 0.305611260672685 |
| 2 | 0.09817644590881472 |
| 3 | 0.0535602614410278 |
| 4 | 0.03338288564939752 |
| 5 | 0.020908131464913212 |
| 6 | 0.012751772822999618 |
| 7 | 0.0076898965693554195 |
| 8 | 0.0047235249707971065 |
| 9 | 0.003064152872041349 |
| 10 | 0.0021541202094981183 |
| 11 | 0.0017016681318381601 |
| 12 | 0.001470641900816825 |
| 13 | 0.0013876234825638389 |
| 14 | 0.0013932954077710861 |
| 15 | 0.001478000686015844 |
| 16 | 0.001628612376623863 |
| 17 | 0.001856349796721636 |
| 18 | 0.002118248961954872 |
| 19 | 0.002493541346501074 |
| 20 | 0.002933296689390702 |
| 21 | 0.003504457293518899 |
| 22 | 0.004190697976951823 |
| 23 | 0.005035599729879704 |
| 24 | 0.006078271665333339 |
| 25 | 0.007325625380795771 |
| 26 | 0.008833168759083703 |
| 27 | 0.010587418241470881 |
| 28 | 0.01264330432916605 |
| 29 | 0.01501480305344328 |
| 30 | 0.017683608020969084 |
| 31 | 0.02066887471567689 |
| 32 | 0.02386984438638007 |
| 33 | 0.02729073418305806 |
| 34 | 0.030854961794205032 |
| 35 | 0.034422489537483544 |
| 36 | 0.03790665922626915 |
| 37 | 0.041241190848334566 |
| 38 | 0.04424662061448773 |
| 39 | 0.046904619488978956 |
| 40 | 0.04908745594256543 |
| 41 | 0.050652301615137584 |
| 42 | 0.05157798584773887 |
| 43 | 0.05186447467674472 |
| 44 | 0.05152110243815013 |
| 45 | 0.05043535896686638 |
| 46 | 0.048878313606605016 |
| 47 | 0.04673832792529317 |
| 48 | 0.04416799482493853 |
| 49 | 0.041235915165407276 |
| 50 | 0.03811378638720565 |
| 51 | 0.034809657869220266 |
| 52 | 0.031422556217813695 |
| 53 | 0.02807980539874134 |
| 54 | 0.024847215594098426 |
| 55 | 0.02175632768936863 |
| 56 | 0.01889390176204846 |
| 57 | 0.016238591674456862 |
| 58 | 0.01386066816464046 |
| 59 | 0.011727701632287599 |
| 60 | 0.0098881468476317 |
| 61 | 0.00827874090037727 |
| 62 | 0.0069244244117450116 |
| 63 | 0.005768557378118415 |
| 64 | 0.0048317727010747675 |
| 65 | 0.00408654852396966 |
| 66 | 0.0034799072539741602 |
| 67 | 0.003020549239435122 |
| 68 | 0.0026761694133288203 |
| 69 | 0.002425885807502049 |
| 70 | 0.0022578507777843543 |
| 71 | 0.002150078538242655 |
| 72 | 0.0021003727745252452 |
| 73 | 0.0021013011135811274 |
| 74 | 0.002133102386848907 |
| 75 | 0.0021830232535182802 |
| 76 | 0.002266234132307241 |
| 77 | 0.0023527960886637438 |
| 78 | 0.002448488599271044 |
| 79 | 0.0025463094969820615 |
| 80 | 0.002659402704283054 |
| 81 | 0.0027581066562179673 |
| 82 | 0.0028576880017727392 |
| 83 | 0.002939596941641869 |
| 84 | 0.0030087072558687023 |
| 85 | 0.0030706116212044802 |
| 86 | 0.0031266346189850504 |
| 87 | 0.0031589227041967645 |
| 88 | 0.003182923665153581 |
| 89 | 0.003177070600618357 |
| 90 | 0.003161152982172472 |
| 91 | 0.003121511772365738 |
| 92 | 0.003072111681264279 |
| 93 | 0.0030167566347556237 |
| 94 | 0.002941866843845564 |
| 95 | 0.0028551294087650813 |
| 96 | 0.0027582255289019474 |
| 97 | 0.002640694408065546 |
| 98 | 0.0025339297560357144 |
| 99 | 0.0024188666585429836 |C
### Chart:
| Category | NERICA 3 |
|---|---|
| 0 | 0.0 |
| 1 | 0.2924356460078034 |
| 2 | 0.07613323867826476 |
| 3 | 0.031482897569720884 |
| 4 | 0.016078878068400852 |
| 5 | 0.0095579085961664 |
| 6 | 0.006415594528715277 |
| 7 | 0.004816441478896347 |
| 8 | 0.004009764614690563 |
| 9 | 0.003594770908648406 |
| 10 | 0.003517529384982622 |
| 11 | 0.003701561110895226 |
| 12 | 0.0041603385250510464 |
| 13 | 0.0048651717223688554 |
| 14 | 0.005886425168328056 |
| 15 | 0.0072480414681030236 |
| 16 | 0.009041359771132148 |
| 17 | 0.01133660026894698 |
| 18 | 0.014149447600747742 |
| 19 | 0.017576208061895755 |
| 20 | 0.021615974787575362 |
| 21 | 0.026325584843096727 |
| 22 | 0.03157400313276726 |
| 23 | 0.03727606680615878 |
| 24 | 0.043332577218762734 |
| 25 | 0.04944342394824615 |
| 26 | 0.05539873649163388 |
| 27 | 0.060931965294965015 |
| 28 | 0.06591501570301007 |
| 29 | 0.06981900003308959 |
| 30 | 0.07262716876686336 |
| 31 | 0.07416468936014901 |
| 32 | 0.07438753764825255 |
| 33 | 0.0733263134485188 |
| 34 | 0.07095484134467375 |
| 35 | 0.06753642129175592 |
| 36 | 0.06324174998077237 |
| 37 | 0.05818078477063771 |
| 38 | 0.05274768895738294 |
| 39 | 0.04713688199117389 |
| 40 | 0.04153121392558747 |
| 41 | 0.036031051337092335 |
| 42 | 0.030930252776943137 |
| 43 | 0.026220010664125746 |
| 44 | 0.02198490388308824 |
| 45 | 0.01823865219926356 |
| 46 | 0.01507080164302255 |
| 47 | 0.01245228444894391 |
| 48 | 0.010269728774678535 |
| 49 | 0.008514251467144449 |
| 50 | 0.007132505516534247 |
| 51 | 0.006095766666826696 |
| 52 | 0.005341090255580654 |
| 53 | 0.004818406352663882 |
| 54 | 0.004473907645673135 |
| 55 | 0.004287821733548991 |
| 56 | 0.004199326841941853 |
| 57 | 0.004185799441773054 |
| 58 | 0.004232248782830125 |
| 59 | 0.00432324442286865 |
| 60 | 0.00442182475045739 |
| 61 | 0.004518721881986551 |
| 62 | 0.004619164030522874 |
| 63 | 0.004716473373331929 |
| 64 | 0.0047752134807180924 |
| 65 | 0.0048043293374552005 |
| 66 | 0.004821442975759183 |
| 67 | 0.004806417874606708 |
| 68 | 0.00475158690419165 |
| 69 | 0.004672703405594258 |
| 70 | 0.004550475890915056 |
| 71 | 0.0044035019590657086 |
| 72 | 0.004249115094366113 |
| 73 | 0.004081626781153643 |
| 74 | 0.0038713784178391786 |
| 75 | 0.0036746231037535766 |
| 76 | 0.003447535909655123 |
| 77 | 0.003240166155112024 |
| 78 | 0.003025039958318743 |
| 79 | 0.002819415231527218 |
| 80 | 0.0026191011600584495 |
| 81 | 0.0024280343616355114 |
| 82 | 0.0022471835327661583 |
| 83 | 0.002094521085254141 |
| 84 | 0.001938533466750925 |
| 85 | 0.0018034243495747755 |
| 86 | 0.0016769922798405914 |
| 87 | 0.00157918141330765 |
| 88 | 0.0014967735082670821 |
| 89 | 0.0014162342943620698 |
| 90 | 0.0013540178718487502 |
| 91 | 0.001298328127933875 |
| 92 | 0.001260851252402708 |
| 93 | 0.001221739272127927 |
| 94 | 0.0011990607815452797 |
| 95 | 0.0011716418612437477 |
| 96 | 0.001159735825442553 |
| 97 | 0.0011438107296623088 |
| 98 | 0.0011304825649454543 |
| 99 | 0.0011211253688916541 |Figure S1
Figure S1. k-mer analysis (k-merlength = 17). The X-axis shows k-mer depth, and the Y-axis shows proportion that
represents the frequency at that k-mer depth divided by to total frequency of all k-mer depth. (A) WAB56-104, (B) CG14,
(C) NERICA 3, (D) NERICA 4, (E) NERICA 5, (F) NERICA 7. Arrow indicates a peak of heterogeneous sequences.Peak
between 0 to 8 of k-mer depth would be due to sequencing error or contaminants.

## Slide 2
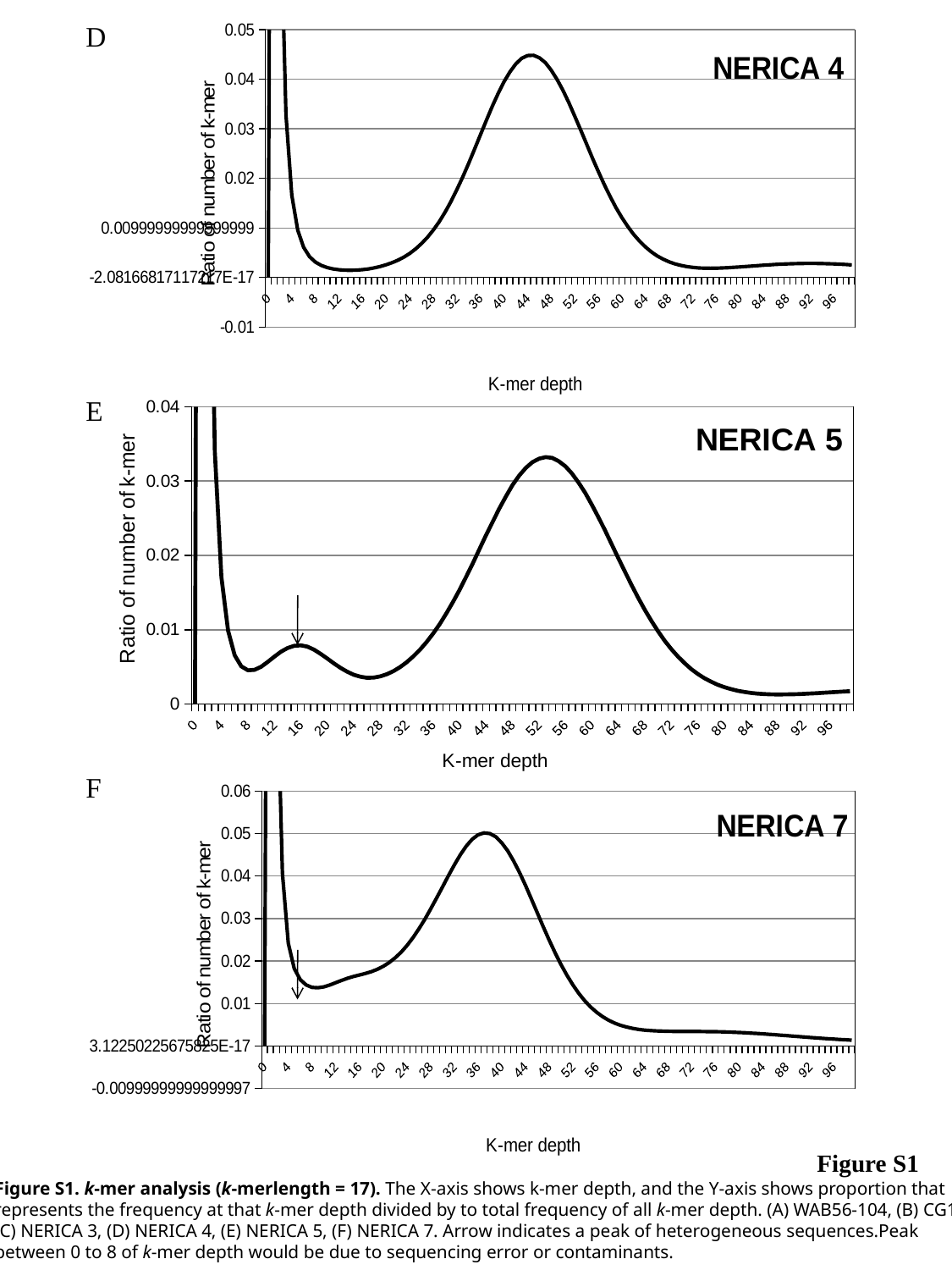

D
### Chart:
| Category | NERICA 4 |
|---|---|
| 0 | 0.0 |
| 1 | 0.2992786329804539 |
| 2 | 0.07939982127348853 |
| 3 | 0.03285833115349499 |
| 4 | 0.0165871795384071 |
| 5 | 0.009551670219217073 |
| 6 | 0.006105949716093411 |
| 7 | 0.004203966151452268 |
| 8 | 0.003107115130513225 |
| 9 | 0.0024392049006042027 |
| 10 | 0.002011124983718756 |
| 11 | 0.0017260475355970475 |
| 12 | 0.001554733580829098 |
| 13 | 0.0014678262862351593 |
| 14 | 0.0014471704310165263 |
| 15 | 0.0014933706767747141 |
| 16 | 0.001581149094014928 |
| 17 | 0.001730264794055226 |
| 18 | 0.001947707255069316 |
| 19 | 0.002222315858683054 |
| 20 | 0.002570472834897409 |
| 21 | 0.0029845379256794214 |
| 22 | 0.003485582050567408 |
| 23 | 0.004107076816182086 |
| 24 | 0.004857277390659743 |
| 25 | 0.005761097881684083 |
| 26 | 0.0068406801772232404 |
| 27 | 0.008098760627236768 |
| 28 | 0.009554934367015173 |
| 29 | 0.01124176601077688 |
| 30 | 0.01317491526915154 |
| 31 | 0.015318988941445736 |
| 32 | 0.017727596940161947 |
| 33 | 0.020280990646941346 |
| 34 | 0.02301400471913353 |
| 35 | 0.02588766922894455 |
| 36 | 0.028794251926283248 |
| 37 | 0.03169413210349648 |
| 38 | 0.034515360154252044 |
| 39 | 0.037111864183520825 |
| 40 | 0.039487769213245295 |
| 41 | 0.04147279638733158 |
| 42 | 0.0430844245254647 |
| 43 | 0.044208413578960895 |
| 44 | 0.044774171867966034 |
| 45 | 0.0448181379403493 |
| 46 | 0.044311979813102496 |
| 47 | 0.043342158970994776 |
| 48 | 0.041833200323102504 |
| 49 | 0.03992878273930259 |
| 50 | 0.03775645394776367 |
| 51 | 0.03524216339930068 |
| 52 | 0.03253928504641914 |
| 53 | 0.029768674345663423 |
| 54 | 0.026943254657704016 |
| 55 | 0.02409239818847319 |
| 56 | 0.021385179595112592 |
| 57 | 0.0187551988349723 |
| 58 | 0.01634068769926485 |
| 59 | 0.01407540499711654 |
| 60 | 0.012065976140432607 |
| 61 | 0.010275286180230454 |
| 62 | 0.008680962637253223 |
| 63 | 0.007316943424949904 |
| 64 | 0.006148542489245826 |
| 65 | 0.005162810848216521 |
| 66 | 0.00433653564546404 |
| 67 | 0.0036940365755721923 |
| 68 | 0.003157670989502835 |
| 69 | 0.0027494270476983627 |
| 70 | 0.002434311241032449 |
| 71 | 0.0022023210318254213 |
| 72 | 0.002044412117308508 |
| 73 | 0.0019318681956595367 |
| 74 | 0.001881788891999958 |
| 75 | 0.0018690038861030327 |
| 76 | 0.0018814968097009095 |
| 77 | 0.0019245661381833417 |
| 78 | 0.0019790061793252016 |
| 79 | 0.0020543941579847535 |
| 80 | 0.0021323493863254 |
| 81 | 0.0022149881799525487 |
| 82 | 0.002302992064230622 |
| 83 | 0.002388879632903319 |
| 84 | 0.0024765196953703003 |
| 85 | 0.002553076003226041 |
| 86 | 0.00262321674900446 |
| 87 | 0.00269007797423214 |
| 88 | 0.002745752959831385 |
| 89 | 0.002785588886020841 |
| 90 | 0.002818983628878657 |
| 91 | 0.0028358065444536503 |
| 92 | 0.0028389425859802714 |
| 93 | 0.0028377127657737552 |
| 94 | 0.0028159551966201067 |
| 95 | 0.0027787172656169325 |
| 96 | 0.0027354993338595326 |
| 97 | 0.0026769137737715416 |
| 98 | 0.0026144696527855946 |
| 99 | 0.002530934115257864 |E
### Chart:
| Category | NERICA 5 |
|---|---|
| 0 | 0.0 |
| 1 | 0.3142687603678507 |
| 2 | 0.08322231876275456 |
| 3 | 0.0338753363350802 |
| 4 | 0.017040127730999062 |
| 5 | 0.009884973328544055 |
| 6 | 0.006571090137187232 |
| 7 | 0.005070785972605683 |
| 8 | 0.0045420379662919445 |
| 9 | 0.004602524781223989 |
| 10 | 0.005019345458132582 |
| 11 | 0.005668897639114429 |
| 12 | 0.006381282937741019 |
| 13 | 0.007038947243101748 |
| 14 | 0.007539561341888374 |
| 15 | 0.007839614353795633 |
| 16 | 0.007901647990516042 |
| 17 | 0.007720500386081626 |
| 18 | 0.007313008434958483 |
| 19 | 0.006747332230950092 |
| 20 | 0.006119770120392138 |
| 21 | 0.005472603347168162 |
| 22 | 0.004864372163959698 |
| 23 | 0.004351644191167594 |
| 24 | 0.003941332331784189 |
| 25 | 0.0036756265845876252 |
| 26 | 0.0035321154136667148 |
| 27 | 0.003555621890843805 |
| 28 | 0.003722795959122655 |
| 29 | 0.004014858506791705 |
| 30 | 0.004428694165249171 |
| 31 | 0.0049677398334685525 |
| 32 | 0.005632073721540356 |
| 33 | 0.006415187011939325 |
| 34 | 0.00731578489316848 |
| 35 | 0.008340489152883686 |
| 36 | 0.009491176833254957 |
| 37 | 0.010775403898018267 |
| 38 | 0.01220616625719879 |
| 39 | 0.01374970756676912 |
| 40 | 0.01538521959767164 |
| 41 | 0.017153028341529465 |
| 42 | 0.018950322289476705 |
| 43 | 0.020834325157672813 |
| 44 | 0.022731936581602465 |
| 45 | 0.024507822689798855 |
| 46 | 0.02631044361390489 |
| 47 | 0.027923000983268404 |
| 48 | 0.029459438209625242 |
| 49 | 0.030724104061772766 |
| 50 | 0.03175728542628879 |
| 51 | 0.0325338203790822 |
| 52 | 0.03299868812154052 |
| 53 | 0.03320980757062709 |
| 54 | 0.033102916102044036 |
| 55 | 0.03264781158587369 |
| 56 | 0.031958576474012054 |
| 57 | 0.030981072003867183 |
| 58 | 0.02976001696714034 |
| 59 | 0.0283791960956998 |
| 60 | 0.026777657659076026 |
| 61 | 0.025085465037641932 |
| 62 | 0.023317886579050352 |
| 63 | 0.021449973264456405 |
| 64 | 0.019595916170882167 |
| 65 | 0.017776614772490932 |
| 66 | 0.015983687554592313 |
| 67 | 0.014272511606210701 |
| 68 | 0.012653671359583297 |
| 69 | 0.01116006284774294 |
| 70 | 0.009777191133931303 |
| 71 | 0.008508580017222468 |
| 72 | 0.007387681691292213 |
| 73 | 0.006378254469239647 |
| 74 | 0.005502757682031199 |
| 75 | 0.004713165989142192 |
| 76 | 0.004045838392609209 |
| 77 | 0.0034814743801179293 |
| 78 | 0.003012292047671245 |
| 79 | 0.002602749254177016 |
| 80 | 0.00227477958499381 |
| 81 | 0.0020126845369717685 |
| 82 | 0.001791771446562382 |
| 83 | 0.0016312669609946015 |
| 84 | 0.0014999478741696101 |
| 85 | 0.0014109795513085961 |
| 86 | 0.001347994358488056 |
| 87 | 0.0013098278343617136 |
| 88 | 0.0012954936627892085 |
| 89 | 0.0012994649973806778 |
| 90 | 0.001317635808388924 |
| 91 | 0.001335167903658718 |
| 92 | 0.0013723133516045458 |
| 93 | 0.00141842254491384 |
| 94 | 0.0014644839431678742 |
| 95 | 0.0015192483864839626 |
| 96 | 0.0015769283281708121 |
| 97 | 0.001628247182503279 |
| 98 | 0.0016778497598514558 |
| 99 | 0.001733613554322945 |F
### Chart:
| Category | NERICA 7 |
|---|---|
| 0 | 0.0 |
| 1 | 0.33980463055715376 |
| 2 | 0.09396011409448661 |
| 3 | 0.04071956944924665 |
| 4 | 0.02415738148888635 |
| 5 | 0.0182221743890827 |
| 6 | 0.015639604800434238 |
| 7 | 0.014363793188995999 |
| 8 | 0.01378684937552076 |
| 9 | 0.013704726667314617 |
| 10 | 0.013914838916431301 |
| 11 | 0.01435336890883592 |
| 12 | 0.01490563237988822 |
| 13 | 0.015428782325636287 |
| 14 | 0.01593834061379102 |
| 15 | 0.016365008691793723 |
| 16 | 0.01669903813940678 |
| 17 | 0.017068000380809037 |
| 18 | 0.01749352293351942 |
| 19 | 0.018034153595228242 |
| 20 | 0.01874331393794268 |
| 21 | 0.01962300290329783 |
| 22 | 0.02074063465474652 |
| 23 | 0.02203807378370381 |
| 24 | 0.023622759107335634 |
| 25 | 0.025458033816288427 |
| 26 | 0.027524601537012798 |
| 27 | 0.029769258347307183 |
| 28 | 0.03224484160127981 |
| 29 | 0.03482323575023758 |
| 30 | 0.03742439721438028 |
| 31 | 0.0400694380248453 |
| 32 | 0.04260955444616028 |
| 33 | 0.044988821933554794 |
| 34 | 0.04703612191886253 |
| 35 | 0.04863037237868524 |
| 36 | 0.049699691600930636 |
| 37 | 0.050146217359875504 |
| 38 | 0.04998735018471057 |
| 39 | 0.04921531768632733 |
| 40 | 0.0477878270709336 |
| 41 | 0.04589539637752059 |
| 42 | 0.04350234249323306 |
| 43 | 0.040793347005698324 |
| 44 | 0.03775752169701238 |
| 45 | 0.03455125940770939 |
| 46 | 0.03128962815340209 |
| 47 | 0.0280255683854752 |
| 48 | 0.02486127814288258 |
| 49 | 0.02189250042955617 |
| 50 | 0.01912463639724903 |
| 51 | 0.0165878878202933 |
| 52 | 0.014335521624781643 |
| 53 | 0.012338939771791308 |
| 54 | 0.01060635567766837 |
| 55 | 0.009148474276269327 |
| 56 | 0.007909170555897203 |
| 57 | 0.0068975056216142505 |
| 58 | 0.006074514430602316 |
| 59 | 0.005395962523698577 |
| 60 | 0.004874290305577611 |
| 61 | 0.004475303846263739 |
| 62 | 0.004166034927844078 |
| 63 | 0.003922650896612042 |
| 64 | 0.00374731106774362 |
| 65 | 0.0036203582272226254 |
| 66 | 0.003541408624076155 |
| 67 | 0.0034947685617994903 |
| 68 | 0.003454005044272404 |
| 69 | 0.003432486351656235 |
| 70 | 0.003422463005348461 |
| 71 | 0.003418740048148441 |
| 72 | 0.003414507332193329 |
| 73 | 0.0034015113477520085 |
| 74 | 0.0033985329819919766 |
| 75 | 0.003372518102603457 |
| 76 | 0.003360541635672288 |
| 77 | 0.003331267736827141 |
| 78 | 0.003291317542257612 |
| 79 | 0.0032327239235556003 |
| 80 | 0.0031721542737243656 |
| 81 | 0.0031084401569657183 |
| 82 | 0.003021397417629043 |
| 83 | 0.00294550636701306 |
| 84 | 0.0028554165303988177 |
| 85 | 0.002756002117905124 |
| 86 | 0.0026561008571622034 |
| 87 | 0.002548238195637668 |
| 88 | 0.0024325115029813892 |
| 89 | 0.00231872647569557 |
| 90 | 0.002212198351136593 |
| 91 | 0.002105956607900684 |
| 92 | 0.0019967078607724567 |
| 93 | 0.001896989884076297 |
| 94 | 0.0017965502264459753 |
| 95 | 0.0017050972147338761 |
| 96 | 0.001622928685516008 |
| 97 | 0.00153049624967898 |
| 98 | 0.0014552237827208548 |
| 99 | 0.0013831072379430667 |Figure S1
Figure S1. k-mer analysis (k-merlength = 17). The X-axis shows k-mer depth, and the Y-axis shows proportion that
represents the frequency at that k-mer depth divided by to total frequency of all k-mer depth. (A) WAB56-104, (B) CG14,
(C) NERICA 3, (D) NERICA 4, (E) NERICA 5, (F) NERICA 7. Arrow indicates a peak of heterogeneous sequences.Peak
between 0 to 8 of k-mer depth would be due to sequencing error or contaminants.
