# Supplementary material for: Comparative whole genome re-sequencing analysis in upland New Rice for Africa: insights into the breeding history and respective genome compositions
Source: Rice (N Y). 2018 May 15;11:33. doi: 10.1186/s12284-018-0224-3 (PMC5953909; doi:10.1186/s12284-018-0224-3)
Supplement: Supplementary file 9 — Figure S2. Phylogeny of genes for fucogalactoxyloglucan biosynthesis. Predicted proteins sequences were aligned by ‘CLUSTAL Ω’ and a phylogenetic dendrogram was constructed by ‘Simple Phylogeny’ using the neighbor-joining method with the option of ‘exclude gaps’. Twelve fucosyltransferase genes in Arabidopsis (AtFUT1 to AtFUT12), four candidate genes for galactosyltransferase in rice, four candidate genes for acetyltransferase genes in rice were analyzed together with the five fucosyltransferase genes with CG14-alleles. The five fucosyltransferase genes of interest were marked with a circle. (PPTX 85 kb) [file 12284_2018_224_MOESM9_ESM.pptx]

## Slide 1
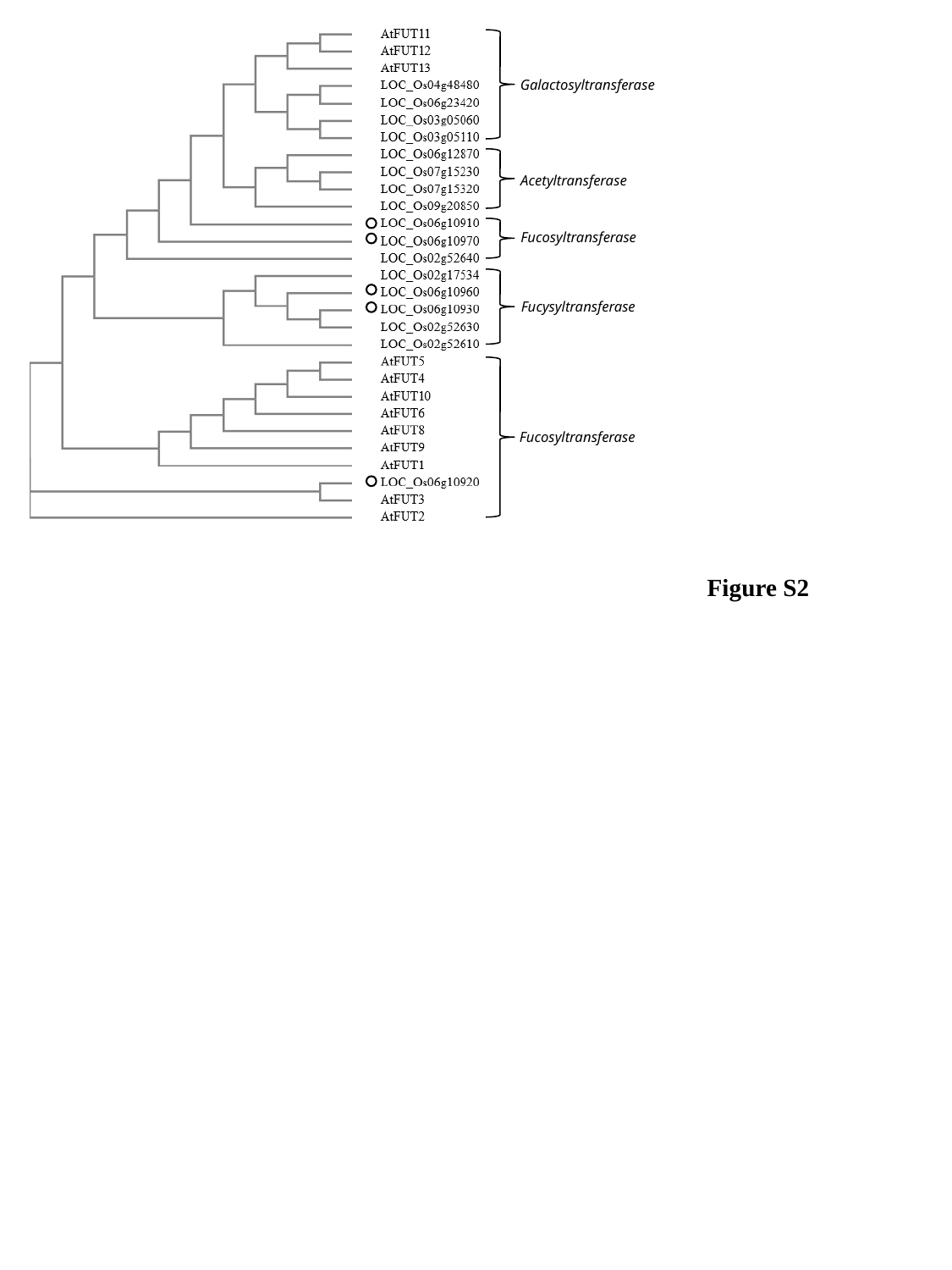

Galactosyltransferase
Acetyltransferase
Fucosyltransferase
Fucysyltransferase
Fucosyltransferase
Figure S2
