# Supplementary figures and images for: Comparative whole genome re-sequencing analysis in upland New Rice for Africa: insights into the breeding history and respective genome compositions
Source: Rice (N Y). 2018 May 15;11:33. doi: 10.1186/s12284-018-0224-3 (PMC5953909; doi:10.1186/s12284-018-0224-3)

## Slide 1
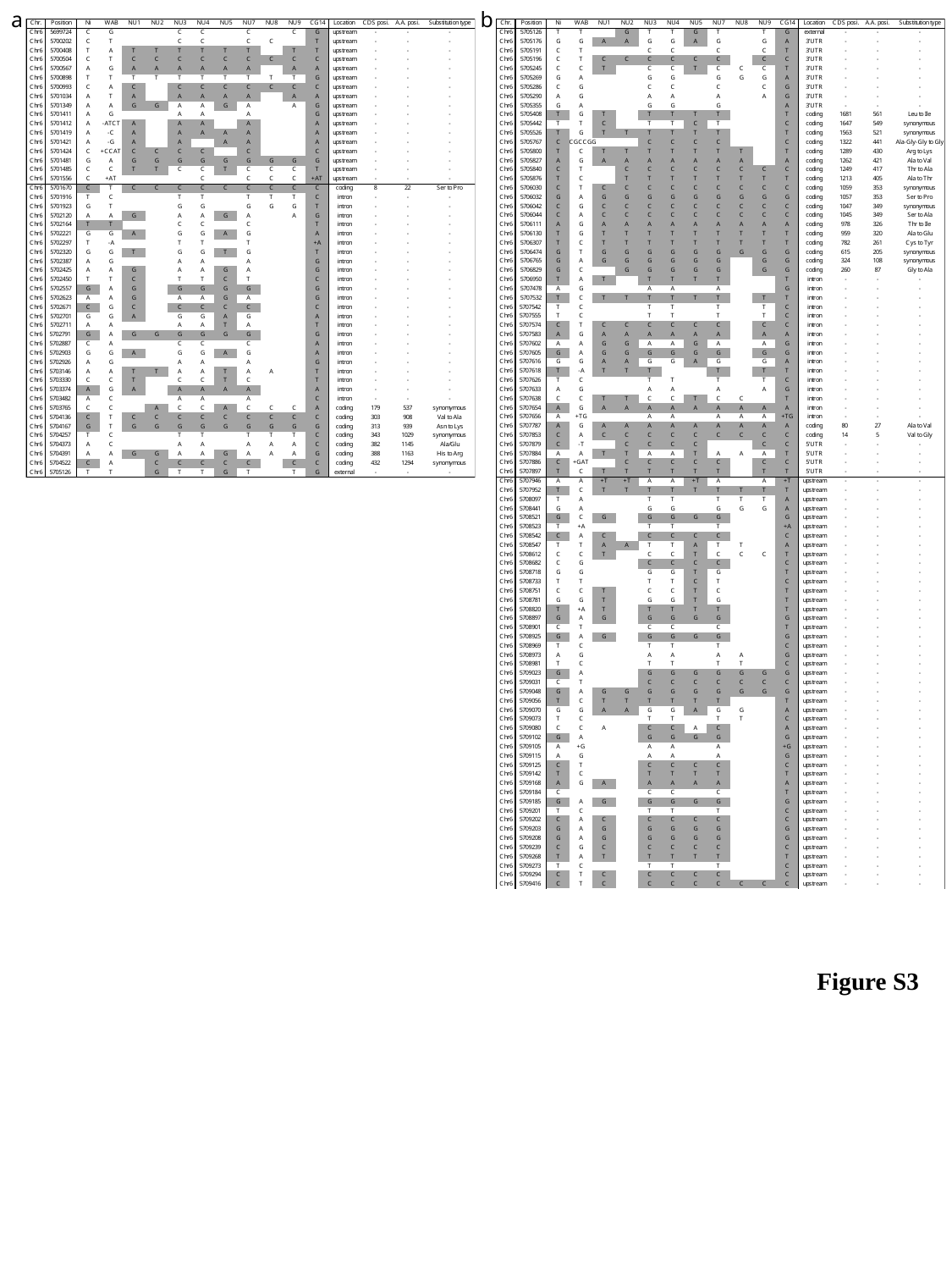

a
b
Figure S3

Supplement: Supplementary file 10 — Figure S3. Graphical representation of allelic patterns in the two fucosyltransferase. Substitution type indicates amino acids of WAB56–104-allele to CG14-allele. (a) LOC_Os06g10910 and (b) LOC_Os06g10920. Varietal names are abbreviated as follows: Nipponbare, Ni; WAB56–104, WAB; NERICA 1, NU1; NERICA 2, NU2; NERICA 3, NU3; NERICA 4, NU4; NERICA 5, NU5; NERICA 7, NU7; NERICA 8, NU8; NERICA 9, NU9. CDS posi. and A.A. posi. Represent positions in coding sequences and positions in deduced amino acid sequences, respectively. Alleles of CG14 are colored in gray. (PPTX 205 kb) [file 12284_2018_224_MOESM10_ESM.pptx]

## Slide 1
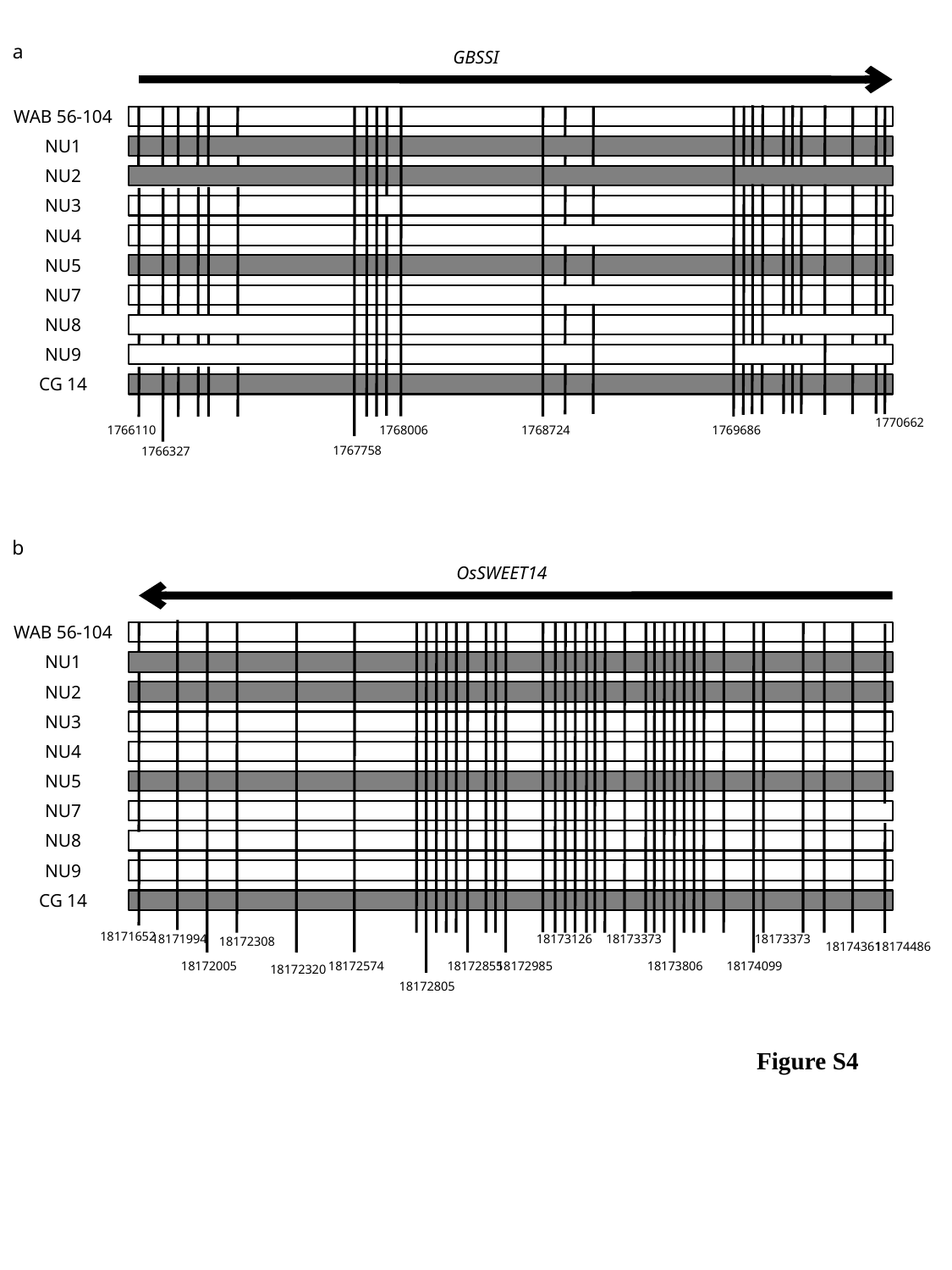

a
GBSSI
WAB 56-104
NU1
NU2
NU3
NU4
NU5
NU7
NU8
NU9
CG 14
1770662
1766110
1768006
1768724
1769686
1767758
1766327
b
OsSWEET14
WAB 56-104
NU1
NU2
NU3
NU4
NU5
NU7
NU8
NU9
CG 14
18171652
18171994
18173126
18173373
18173373
18172308
18174361
18174486
18172005
18172574
18172855
18172985
18173806
18174099
18172320
18172805
Figure S4

Supplement: Supplementary file 14 — Figure S4. Graphical genotype of GBSSI and OsSWEET14 at gene level. Polymorphic position is indicated by vertical line. WAB56–104 segment is presented by while rectangle, and CG14 allele is presented in gray rectangle. Arrow represent the position of gene. (a) GBSSI, (b) OsSWEET14. Representative polymorphic sites were used. (PPTX 76 kb) [file 12284_2018_224_MOESM14_ESM.pptx]
